# Age-Related Changes in the Thermoregulatory Properties in Bank Voles From a Selection Experiment

**DOI:** 10.3389/fphys.2020.576304

**Published:** 2020-11-19

**Authors:** Marta Grosiak, Paweł Koteja, Ulf Bauchinger, Edyta T. Sadowska

**Affiliations:** ^1^Institute of Environmental Sciences, Faculty of Biology, Jagiellonian University, Kraków, Poland; ^2^Nencki Institute of Experimental Biology, Polish Academy of Sciences, Warsaw, Poland

**Keywords:** aging, bank vole, body temperature, evolution, metabolic rate, thermal conductance

## Abstract

As with many physiological performance traits, the capacity of endotherms to thermoregulate declines with age. Aging compromises both the capacity to conserve or dissipate heat and the thermogenesis, which is fueled by aerobic metabolism. The rate of metabolism, however, not only determines thermogenic capacity but can also affect the process of aging. Therefore, we hypothesized that selection for an increased aerobic exercise metabolism, which has presumably been a crucial factor in the evolution of endothermic physiology in the mammalian and avian lineages, affects not only the thermoregulatory traits but also the age-related changes of these traits. Here, we test this hypothesis on bank voles (*Myodes glareolus*) from an experimental evolution model system: four lines selected for high swim-induced aerobic metabolism (A lines), which have also increased the basal, average daily, and maximum cold-induced metabolic rates, and four unselected control (C) lines. We measured the resting metabolic rate (RMR), evaporative water loss (EWL), and body temperature in 72 young adult (4 months) and 65 old (22 months) voles at seven ambient temperatures (13–32°C). The RMR was 6% higher in the A than in the C lines, but, regardless of the selection group or temperature, it did not change with age. However, EWL was 12% higher in the old voles. An increased EWL/RMR ratio implies either a compromised efficiency of oxygen extraction in the lungs or increased skin permeability. This effect was more profound in the A lines, which may indicate their increased vulnerability to aging. Body temperature did not differ between the selection and age groups below 32°C, but at 32°C it was markedly higher in the old A-line voles than in those from other groups. As expected, the thermogenic capacity, measured as the maximum cold-induced oxygen consumption, was decreased by about 13% in the old voles from both selection groups, but the performance of old A-line voles was the same as that of the young C-line ones. Thus, the selection for high aerobic exercise metabolism attenuated the adverse effects of aging on cold tolerance, but this advantage has been traded off by a compromised coping with hot conditions by aged voles.

## Introduction

Aging is one of the most challenging issues in medicine and biomedical research (Wahlich et al., 2019) and is a puzzling phenomenon within evolutionary–ecology (review in Flouris and Piantoni, 2015). Aging can be defined as a time-dependent persistent decline in functionality and reproducibility (Höhn et al., 2017; Delbaere et al., 2020). It occurs in all higher organisms (Kirkwood and Austad, 2000; Höhn et al., 2017) and across all levels of organization from the molecular to organismal performance (Troen, 2003; Khan et al., 2017; Luceri et al., 2018). Aging compromises an animal’s thermoregulatory capability (e.g., Kenney and Hodgson, 1987; Kenney and Munce, 2003; DeGroot and Kenney, 2007; Hanna and Tait, 2015) through the loss of the efficiency of the thermoregulatory behavior in ectotherms and a decline of the cellular metabolism in humans and other homeothermic or heterothermic endotherms such as birds and mammals (Flouris and Piantoni, 2015). The core of endothermic physiology is the ability to thermoregulate through a controlled increase of metabolic heat production, i.e., active thermogenesis (Van Sant and Hammond, 2008; Seebacher, 2009; Cannon and Nedergaard, 2010). However, the evolution of the endothermic strategy of birds and mammals has also been tightly related to the evolution of high rates of aerobic metabolism at its basal, average daily, and maximum exercise levels (Bozinovic, 1992; Hayes and Garland, 1995; Koteja, 2004; Sadowska et al., 2005; Swanson et al., 2012; review in Legendre and Davesne, 2020). The network of biochemical processes associated with aerobic metabolism is recognized as an important factor behind aging at the cellular level, and, since the seminal work of Denham Harman (1956), the hypothesis linking a high rate of metabolism with accelerated aging has been subject to a vivid and continuing debate (Holloszy and Smith, 1986; Speakman et al., 2004; Andziak et al., 2006; Brys et al., 2007; Gems and Doonan, 2009; Salmon et al., 2010; Rudolf et al., 2017; Viña, 2019). An intriguing question arises: whether and how selection for an increased level of metabolism would affect the aging of thermoregulatory performance. Surprisingly, despite innumerous studies on the relation between pairs of the three features – the rates of metabolism, thermoregulation, and aging – the question has apparently not yet been addressed. Here, we tackle this question using a unique model of experimental evolution, lines of a common non-laboratory rodent, the bank vole, *Myodes glareolus*, selected for the high rate of aerobic exercise metabolism (Sadowska et al., 2008; Rudolf et al., 2017; Stawski et al., 2017).

With increasing age, both the hypo- and hyperthermia occurrence increases (Kenney and Munce, 2003). Elderly people have altered responses to changes in body temperature and are unable to regulate their body temperatures as efficiently as young adults (Güneş and Zaybak, 2008). Several studies reported that body temperature generally decrease in older people (DeGroot and Kenney, 2007; Güneş and Zaybak, 2008; Lu et al., 2009), and a similar pattern has also been shown in various animal models, such as laboratory mice and rats (Balmagiya and Rozovski, 1983; Talan and Engel, 1986; Scarpace et al., 1994; Gonzales and Rikke, 2010). The decreased body temperature in aged individuals can at least be partly due to the age-related decline of the basal metabolic rate (BMR) (review in De Magalhães et al., 2007; Moe et al., 2009) and a decreased thermogenic capacity (Lin et al., 2015). Such adverse changes place the aged individuals at an increased risk of hypothermia at cold ambient temperatures. Indeed, epidemiological data show an increased mortality among the elderly in harsh winter conditions (Laake and Sverre, 1996; Sheth et al., 1999; Keatinge and Donaldson, 2004; Wilkinson et al., 2004; Analitis et al., 2008; Milne, 2017; Chirakijja et al., 2019). Such an increased winter mortality of old individuals is also evident in population ecology studies on wild animals (Gaillard et al., 2000; Patil et al., 2013). Thus, it can be hypothesized that the selection for a high rate of metabolism could attenuate the adverse effects of aging on the cold response aspect of thermoregulatory performance, provided that the increased metabolism is also persistent at old age.

However, aged individuals are susceptible not only to low but also to high ambient temperatures. Epidemiological studies show an increased mortality among the elderly also during the periods of hot weather (Foster et al., 1976; Fish et al., 1985; Worfolk, 2000; Havenith, 2005; Stapleton et al., 2015; Cheng et al., 2018; Perčič et al., 2018), which – in the face of global climate warming – has recently become a hot issue (Krstič, 2011; review in Balmain et al., 2018; Cheng et al., 2018; Huang et al., 2019). The increased risk of hyperthermia with increased age was also observed in studies on animal models, for instance in horses (McKeever et al., 2010). The increased vulnerability of aged individuals, despite their typically decreased rate of metabolic heat production, is a consequence of both a decreased capacity to dissipate excess heat, which leads to hyperthermia (Stapleton et al., 2015), and an increased sensitivity to the adverse effects of hyperthermia (Goldstein et al., 2003; Szekely and Garai, 2018). The age-related impairment in thermosensitivity of the evaporative heat loss response (Stapleton et al., 2015), decreased sweating response, and changes in skin vasodilatation (leading to impaired heat loss rate) (Armstrong and Kenney, 1993; Gagnon et al., 2017) together limits the defense mechanisms and causes hyperthermia (Szekely and Garai, 2018). An increased basal rate of metabolism should certainly escalate such consequences of the inability to dissipate the excess heat. Thus, it can be hypothesized that the selection for a high rate of metabolism could exacerbate the age-related decline of the ability to cope with high ambient temperatures, even if the increased aerobic metabolism is not associated with accelerated aging at the biochemical or cellular level. However, a selection for a high rate of metabolism can also result in an increased thermal conductance (as a correlated response to the selection) and, hence, increased heat loss (Taylor et al., 2013), as has also been shown in our earlier study on bank voles (Stawski et al., 2017). Thus, the predictions concerning the effects of selection on coping with hot conditions are less straightforward than those concerning coping with cold.

In this study, we asked how the selection for a high metabolism affects the aging of the thermoregulatory performance using the experimental evolution approach (Swallow et al., 2009). This method has been applied in a few previous studies that have targeted the link between thermoregulation and metabolic rate. A study performed on mice selected for high and low heat loss revealed that the selection affected many traits linked to aerobic metabolism (Mousel et al., 2001). The high-heat-loss mice had higher locomotor-related heat loss, food intake, and body temperature than the mice selected for low heat loss and the control mice (Mousel et al., 2001), but not a higher thermogenesis in brown adipose tissue (BAT) (McDaneld et al., 2002). In another experiment, mice selected for low BMR had lower swim- and heliox-induced levels of hypothermia and also larger BAT mass than mice selected for high BMR, which suggests a negative genetic correlation between these traits and BMR (Ksia̧żek et al., 2004). However, when mice were exposed to high temperatures, the animals selected for high BMR had a shorter time to reach the maximum body temperature than the mice selected for lower BMR (Gȩbczyński, 2005). The thermoregulation curves of both selected groups were mostly parallel, but the high-BMR mice had decreased lower critical temperature in comparison to the low-BMR mice (Gȩbczyński, 2005). In these studies, however, the question of how the thermoregulatory parameters changed with age was not addressed.

Here, we used lines of the bank vole selected for a high swim-induced rate of aerobic metabolism (A lines) and unselected control (C) lines (Sadowska et al., 2008, 2015). Voles from the A lines achieved about 60% higher swim-induced rates of oxygen consumption than those from the C lines (Jaromin et al., 2019) and had higher maximum run-induced aerobic metabolic rates (Jaromin et al., 2019). Moreover, voles from the selected lines had higher mass-corrected basal metabolic rates (Sadowska et al., 2015). The thermogenic capacity, measured as the maximum cold-induced rate of oxygen consumption ($\dot{V}$_*O*__2__*cold*_), was higher in the A than in the C lines (Dheyongera et al., 2016; Stawski et al., 2017), although the non-shivering thermogenic capacity was not affected by the selection (Stawski et al., 2015). The selection increased cold tolerance, but reduced the capacity to thermoregulate at high environmental temperatures (Stawski et al., 2017). Contrary to the main assumption of the oxidative stress theory of aging (Denham Harman, 1956; review in Cedikova et al., 2016), the oxidative damage markers were not increased and the antioxidant activity remained unchanged in animals from the A and C lines even if the animals’ energy expenditure was elevated during reproduction (Ołdakowski et al., 2012, 2015). In our previous study on the effects of aging, the maximum run-induced aerobic metabolic rate decreased with age in the A lines, whereas it remained constant in the C lines (Rudolf et al., 2017). To summarize, we know that the selection for a high aerobic metabolism inflicts changes in the thermoregulatory traits and affects the pattern of age-related changes in some locomotor performance traits. In the present study, we asked whether and how the selection affected age-related changes of the thermoregulatory parameters.

## Materials and Methods

### Animal Model and the Selection Experiment

The study was performed on bank voles (*M. glareolus* Schreber, 1780) from four replicate lines selected for the maximum swim-induced rate of aerobic metabolism (A lines: high aerobic metabolism) and four replicate lines of control, randomly bred voles (C lines: control). The selection criterion is the highest 1-min rate of oxygen consumption achieved during an 18-min swimming trial, adjusted for differences in body mass and other confounding factors, such as sex and measurement date. The swimming trial is performed at a water temperature of 38°C, which is close to the voles’ body temperature (*T*_*b*_) to ensure that the increase of metabolic rate is due to exercise, not due to thermoregulation (Jaromin et al., 2016). In the 22nd generation, the mass-adjusted maximum rate of oxygen consumption during swimming was about 60% higher in the A than in the C lines (Jaromin et al., 2019). Four replicate selected lines (A) and four control lines (C) are maintained, with 15–20 reproducing families per line. The details of the colony origin, the selection protocol, animal husbandry, and the results of selection in successive generations are presented in our earlier works (Sadowska et al., 2008, 2015; Lipowska et al., 2019).

This study was conducted on 72 young adult females from generation 27th and 65 old females from 24th generation. The age of the voles from the young group ranged from 93 to 161 days (mean ± SD = 114.4 ± 14.97 days), and voles from the old group were from 605 to 752 days (663.2 ± 22.1 days) old. Within the two age groups, the animals represented both selection directions and all replicate lines within the selection directions. We only used females because this work is a part of an ongoing project concerning limits to energy budgets during lactation (cf. Sadowska et al., 2016). The voles were kept in individual cages (standard polypropylene, 267 mm × 207 mm × 140 mm; Tecniplast 1264, Italy) with sawdust bedding and equipped with coconut shell hut, under controlled ambient temperature (20 ± 1°C) and photoperiod (16:8 h light/dark). Food (standard rodent chow: 24% protein, 3% fat, 4% fiber; Labofeed H, Kcynia, Poland) and water were provided *ad libitum*. All the animal care and measurement procedures were approved by the II Local Bioethical Committee in Kraków, Poland (No. 292/2018).

### General Scheme of the Used Procedures

The experiment was performed on 137 individuals in three subsequent blocks (48, 48, and 41 individuals). Completing measurements on one block lasted 40 days. On the first day, we placed the voles in individual cages, and after 4 days thermosensitive data loggers were implanted to record the core body temperature. After a recovery period of 7–8 days, the resting metabolic rate (RMR) was measured at 20°C in order to familiarize all the voles with the respirometric measurements. After 3 days, a set of RMR measurements at five ambient temperatures (*T*_*a*_ = 13, 17, 23, 26, and 28°C) was started. The order of the measurements was randomized among individuals (although, for logistical reasons, not completely; see Supplementary Table 1) to allow a methodologically proper separation of the effect of temperature from the effect of the measurement order (in a repeated-measures model; see below). To minimize a possible carryover effect, we kept 3-day intervals between the subsequent measurements. Four days after the last of these trials, we measured the maximum thermogenic capacity (see below). Four days after this procedure, we measured the RMR at 32°C. This measurement was done last as it is potentially detrimental to the animals (Stawski et al., 2017).

### Data Loggers’ Implantation

Each female was implanted with a temperature logger (weight = 1.3 ± 0.1 g, temperature resolution = 0.032°C, nominal accuracy = ± 0.2°C; DST nano-T loggers, Star-Oddi, Garðabær, Iceland). The loggers were programmed to record the *T*_*b*_ every 9 min. Before implanting the loggers in females from the first experimental block, they were calibrated as described in Rintoul and Brigham (2014) with a precise thermometer (resolution = ± 0.1°C, accuracy = ± 0.42°C in the relevant range; Oakton, model 300, Digital Thermometer, Vernon Hills, IL, United States) in a water bath in a temperature range of 30–45°C. As the manufacturer warrants stability of the temperature readings for at least 12 months, the calibration was not repeated before reusing the loggers in the next blocks.

During the surgery, the voles were anesthetized by a controlled inhalation system (Sigma Delta Vaporizer UNO BV, Abingdon, United Kingdom) *via* a nose cone for 7–25 min. Isoflurane (VetPharma, Barcelona, Spain) with oxygen was provided with a flow of 200 ml min^–1^ (in accordance with DLAR Veterinary Medical Staff, 4–6563) at 3% volume of breathing air (Hohlbaum et al., 2017). During the surgery, the animals’ eyes were protected from drying by water, as suggested in Vandebroek et al. (1999), using a wet cotton ear stick. The sterilized data loggers were inserted in the abdominal cavity at the height of the animals’ groins *via* a 1-cm-long incision in the skin and a 0.7-cm-long incision on the peritoneum (Valencak et al., 2013). The loggers were placed between the animals’ intestines, but later they could move slightly inside the lower abdomen area. The wounds were sutured by absorbable sutures (Safil 5/0, Aesculap AG, Tuttlingen, Germany). After implantation, we observed their condition carefully for the next 3 days (Curtis et al., 2001; Stawski et al., 2017). Post-surgical care included re-suturing the opened wound (nine individuals) and using an aluminum spray (Alu-Spray, MEDIVET, Poland) for a faster healing of the slow-healing wound (one individual). Out of the 137 implanted animals, two died after the surgery. After completing measurements on a block of individuals, the data loggers were surgically removed under anesthesia, the body temperature records were collected, and the 48 data loggers were reused in the next block.

### Resting Metabolic Rate and Evaporative Water Loss

The RMR was measured as the minimum rate of oxygen consumption ($\dot{V}$_*O*__2_, in milliliters O_2_ per minute) in fasted animals. We recorded the body mass (MB) before and after each measurement (±0.1 g). We used two experimental setups for the RMR measurements: one for all *T*_*a*_ values ranging from 13 to 28°C and another for *T*_*a*_ = 32°C. In the first setup, the animals were acclimated for 2 h in room temperature (20°C) without food but with water provided *ad libitum* and then placed in the respirometric chambers for further measurements. The measurements were conducted at two time intervals: in the morning (m – the morning group, 8:00–12:30) and in the afternoon (a – the afternoon group, 13:00–17:30), each time for eight individuals.

The second setup was introduced because of a higher risk of mortality at 32°C (Stawski et al., 2017). Therefore, we included video recording that allowed observing the behavior of the voles in real time. We could simultaneously measure and video record two animals. Chambers were separated by a thick cardboard so that the camera recorded two animals, but they could not see each other. Before the measurements, the animals were acclimated for 1.5 h at room temperature (20°C) without food but with water provided *ad libitum*. Eight individuals were measured daily at four time intervals (*1*: 8:00–10:20; *2*: 10:40–12:00; *3*: 12:20–14:40; *4*: 15:00–17:20). The trial started at 28°C, then the temperature was gradually increased to 32°C (+1°C every 5 min) and then maintained constant for 2 h.

The RMR was measured using an eight-channel open-flow respiratory system (FMS, Sable Systems, Las Vegas, NV, United States). We used black-colored glass chambers of about 800 ml fitted with wire baskets. To ensure that the voles could not exhale air directly into the outgoing air, the baskets with animals were suspended 3 cm below the ceiling of the chamber with the air inlet near the bottom and the outlet at the top of the chamber. We used paraffin oil at the bottom of each chamber to collect urine and feces; therefore, baskets were placed 4 cm above the chamber bottom (Belay and Teeter, 1993). The chambers were placed in a temperature-controlled cabinet (PTC-1 Peltier, Sable Systems).

A flow rate of 500 ml min^–1^ of fresh air dried with silica gel (Sigma-Aldrich) was regulated by a pump with an eight-channel mass flow controller (MFS Mass Flow System, Sable Systems). First, the water vapor pressure was measured in the subsamples of air flowing out of the chambers, then the air subsamples were dried by a small-volume magnesium perchlorate absorbent (Anhydrone, J.T. Baker, United States), and the oxygen and CO_2_ concentrations were measured with the integrated respirometry system (FMS, Sable Systems). The air samples were drawn sequentially from an empty reference chamber and eight channels with animals. The channels were switched with a computer-controlled baselining unit and multiplexer (RM-8, Sable Systems). In each measurement cycle, lasting 19 min, the reference and the first active channel were sampled for 150 s, and each of the next seven active channels were sampled for 120 s, which ensured a complete washout of the system after switching channels. The time was longer for the reference and the first measurement channels because the change of the air composition after switching to those channels is larger than that in the case of the other channels (Sadowska et al., 2015; Stawski et al., 2017). In the first setup, about 15–17 cycles were recorded for each vole at each temperature. In the second setup (measurements at 32°C), the same respirometric system as described above was used, but we only used two channels, each repeated interchangeably four times within each of the measurement cycle (each lasting 19 min). Therefore, even though the total time of these trials was shorter, and only seven full cycles were recorded, a total of 28 readings were recorded for each individual.

The data were recorded every second through an analog-to-digital interface UI2 (Sable Systems Int., United States) with the ExpeData 1.9.17 v PRO data acquisition program. To calculate the rate of oxygen uptake ($\dot{V}$*O*_2_) and the rate of CO_2_ production ($\dot{V}$*CO*_2_), the last 20 s of the record before switching channels was used. The adequate baselines were computed by interpolating the oxygen concentration values (in percent) on the recorded baseline in the reference channel. The rate of oxygen consumption was computed using an appropriate respirometric equation (Sadowska et al., 2015). Because RMR estimation is associated with stable readings, we did not use an “instantaneous correction” to the readings. The lowest readings of $\dot{V}\,O$_2_ from the last seven to nine cycles in the first setup and from the last six cycles in the second setup were used to calculate the RMR (in milliliters O_2_ per minute).

Based on the RMR and body temperature recorded at or near the time the RMR was recorded (*T*_*b*_rmr), the thermal conductance (*C*_*T*_, in milliliters O_2_ per minute per degree Celsius) was calculated for each ambient temperature as follows (Lovegrove et al., 1991): *C*_*T*_ = RMR/(*T*_*b*_rmr - *T*_*a*_).

The values of water vapor pressure (WVP, in kilopascals) at the time when the RMR was measured and the mean barometric pressure calculated separately for each measurement were used for the calculation of the rate of the water vapor production ($\dot{V}$_*H_2*__*O*_, in milliliters per minute) and then the evaporative water loss (EWL, in milligrams H_2_O per minute) (Lighton, 2008):

$${\dot{V}}_{H_{2}\,O}=F\,R_{\text{i}}\,\left(F_{e\,H_{2}\,O}-F_{i\,H_{2}\,O}\right)\,/\,\left(1-F_{e\,H_{2}\,O}\right)$$

$$E\,W\,L={\dot{V}}_{H_{2}\,O}\times 0.803$$

where FR_*i*_ is the incoming air flow rate (500 ml min^–1^), *F*_*eH_2*__*O*_ the fraction of the water vapor in excurrent air (WVP/barometric pressure), and *F*_*iH_2*__*O*_ the fraction of the water vapor in incurrent air (WVP/barometric pressure).

Finally, the RMR and EWL measured together were converted to their energy equivalents (*W* = J/s) according to Lighton (2008):

$$R\,M\,R\,\left[\text{W}\right]=R\,M\,R\,\left[\text{ml}\,{\text{O}}_{2}\,\min^{-1}\right]\times \left(16+5.164\times \mathrm{RQ}\right)\,/\,60$$

$$E\,W\,L\,\left[\text{W}\right]=E\,W\,L\,\left[\text{mg}\,{\text{H}}_{2}\,\text{O}\,\min^{-1}\right]\times 2430\,/\,60$$

where RQ is the respiratory quotient; the information was combined in a dimensionless EWL/RMR ratio. The ratio provides information about the proportion of evaporative heat loss (Whitfield et al., 2015; Luna et al., 2020). In addition, assuming that the EWL is mainly due to the respiratory water loss (Muñoz-Garcia et al., 2016), and hence proportional to the rate of ventilation (Dawson et al., 2000), the EWL/RMR ratio also provides a proxy of the efficiency (actually: inefficiency) of oxygen extraction in the lungs.

### Thermogenic Capacity

The thermogenic capacity was measured as the rate of oxygen consumption ($\dot{V}$_*O*__2__*cold*_, in milliliters per minute) in completely soaked individuals placed in wet respirometric chambers (500 ml) maintained at +23°C in a temperature-controlled cabinet for up to 18 min (Sadowska et al., 2005). Before the measurements, body mass was recorded and then the animals were soaked in water maintained at 38°C containing a drop of dog shampoo to ensure complete wetting (Stawski et al., 2017).

We measured two animals simultaneously using two respirometric chambers of 500 ml volume and two separate open-flow respirometric systems. The respirometric chambers were partly filled with warm water (38°C). A pass of about 2,000 ml min^–1^ of fresh air dried with silica gel (Sigma-Aldrich) was regulated (±1%) by a mass flow controller (flow rate, 0–3 L min^–1^; Aalborg GFC171S or GFC17, Orangeburg, NY, United States). The subsample of excurrent air was pre-dried with an ND-2 gas drier (Sable Systems) and then by a small volume of a magnesium perchlorate absorbent (Anhydrone, J.T. Baker). The oxygen and CO_2_ concentrations were measured in dried samples of ∼200 ml min^–1^ of air pull through the analyzers (O_2_: FC10 and FC10a; CO_2_: CA2 and CA2a) by an ND-2 pump. The gas concentrations were recorded every 1 s through an analog-to-digital interface UI2 with the ExpeData 1.9.17 v PRO data acquisition program (Sable Systems Int.). The maximum thermogenic capacity was defined as the highest 1-min instantaneous rate of oxygen consumption (Sadowska et al., 2005). The rates of oxygen consumption were calculated with appropriate respirometric equations and corrected for “effective volume” to achieve “instantaneous” rates (Bartholomew et al., 1981; Lighton, 2008). The effective volumes of two used chambers were: 540 ml for O_2_ and 530 ml for CO_2_, and 510 ml for O_2_ and 500 ml for CO_2_.

### Statistical Analysis

For all analyses of RMR, *T*_*b*_rmr, *C*_*T*_, EWL, and EWL/RMR at all ambient temperatures, we collected a total of 916 records of RMR (together with other variables) for all individuals. Among these records, 47 were excluded from the analyses due to death of animals (10 animals died during the measurements), assumed bad condition of animals during measurements (weak, ruffled fur, seven individuals), or system errors (30 RMR records). The highest mortality was recorded at 32°C, where, despite the shorter trial time and real-time monitoring by video recordings, six individuals died (five from the A lines and one from the C lines). In addition, for the analyses of *T*_*b*_rmr and *C*_*T*_, we excluded 11 missing records because of a malfunction of the data loggers and, for EWL and EWL/RMR analyses, of seven records with system errors. For the analyses of the thermogenic capacity, a total of 127 records were collected.

The size of the respirometric chambers was large enough to allow free movement, and many individuals showed long periods of intensive activity during the measurements carried out under conditions similar to those used in our experiment (Sadowska et al., 2015; Stawski et al., 2017). To check whether the minimum observed value of RMR was taken under resting conditions, we analyzed the variation of $\dot{V}\,O$_2_ within the 20-s period from which the RMR was computed. When the animal is active, the variation is increased. Thus, the standard deviation (SD) of the 20 values can be used as an “activity index.” The distribution of the activity index showed that records with SD over 0.06 ml O_2_ min^–1^ are obvious outliers from the typical distribution, and the individuals had to be active when the minimum rate of oxygen was recorded (Supplementary Figure 1). A total of 36 records with such readings were excluded from further analyses (both of the RMR and the other traits measured together). However, for the remaining records, there was still a considerable variation of the SD values, and it could not be said for a particular record whether a relatively high SD is due to a measurement noise or a not perfect resting state. Therefore, the activity index (SD of the $\dot{V}$_*O*__2_ second-by-second readings) has been included as an additional covariate in all analyses of RMR, *T*_*b*_rmr, *C*_*T*_, EWL, and EWL/RMR.

All statistical analyses were performed in SAS v. 9.4 (SAS Institute, Inc., Cary, NC, United States). For the analyses of MB, RMR, *T*_*b*_rmr, *C*_*T*_, EWL, and EWL/RMR measured at 20 and 32°C and for $\dot{V}$_*O*__2__*cold*_, we used a cross-nested mixed analysis of covariance (ANCOVA) model [mixed procedure with the restricted maximum likelihood (REML) method and Satterthwaite option]. For the analyses of RMR, *T*_*b*_rmr, *C*_*T*_, EWL, and EWL/RMR measured at 13, 17, 23, 26, and 28°C, we used the repeated-measures versions of the ANCOVA model. The results of the measurements at 20 and 32°C were not included in the repeated-measures models because these trials were always performed as the first and the last ones, respectively.

All models included age (young *vs*. old) and selection (A *vs*. C lines) as the main fixed categorical factors and the replicated lines nested within the selection groups as the random effect. Timing (“morning” and “afternoon”) and block (three groups) were included as fixed cofactors. In the analyses of RMR, *C*_*T*_, EWL, and EWL/RMR measured at 32°C, the models also included chambers as the fixed cofactor (two chambers), but in the analyses of these traits measured at other ambient temperatures, the models included chambers as the random effect (eight chambers). For the analysis of $\dot{V}$_*O*__2__*cold*_, timing was not included, but analyzer was an additional cofactor (two respirometric systems). In all models, we also included interactions between the age and selection factors and random interactions between age and line nested within selection. In all models, the mean body mass measured before the RMR measurements and the activity index were included as covariates. To test the assumption of the homogeneity of body mass slopes, all initial, full ANCOVA models included interactions between the main factors (and their interactions) and the initial body mass. These interactions were not significant; therefore, we removed them from the final models. The repeated-measures extension of the above models was used to perform the analyses of RMR, *T*_*b*_rmr, *C*_*T*_, EWL, and EWL/RMR for combined results from measurements at other ambient temperatures. These models included a fixed repeated-measures factor for *T*_*a*_ (order of the measurements treated as a grouping factor) and the interactions of *T*_*a*_ with age, selection, and age × selection interaction.

The identification (ID) number of an animal was included as a random effect. For the RMR analysis, we used a model with an “unstructured” type of residual (co)variance matrix and, for the other response variables, the “compound symmetry” type of residual (co)variance matrix. For the RMR, we performed additional analysis with *T*_*a*_ treated as a quantitative predictor and the interactions of *T*_*a*_ with age, selection and age × selection interaction.

The results of all analyses are expressed as adjusted least squares mean (LSM) ± SD. All presented LSMs were calculated for fixed body mass = 25 g and activity index = 0.03. Before estimating the final models, we analyzed studentized residuals and removed from the final analysis observations with residuals higher than 3.5 and lower than -3.5 as the outliers (Stawski et al., 2017; Supplementary Table 2).

Based on the adjusted RMR and *C*_*T*_ estimated for the lowest measured temperature (13°C) for the Selection × Age subgroups and the respective $\dot{V}$_*O*__2__cold_ values, the lower lethal temperature (LLT, in degree Celsius), i.e., the minimum temperature at which the animals can retain homeothermy, was estimated from the linear equation of the thermoregulatory model (Lovegrove et al., 1991) rearranged as follows:

$$L\,L\,T=13-\left(\dot{V}\,O_{2\,c\,o\,l\,d}-R\,M\,R_{13}\right)\,/\,\left(C\,T_{13}\right)$$

## Results

### Body Mass

**FIGURE 1:**
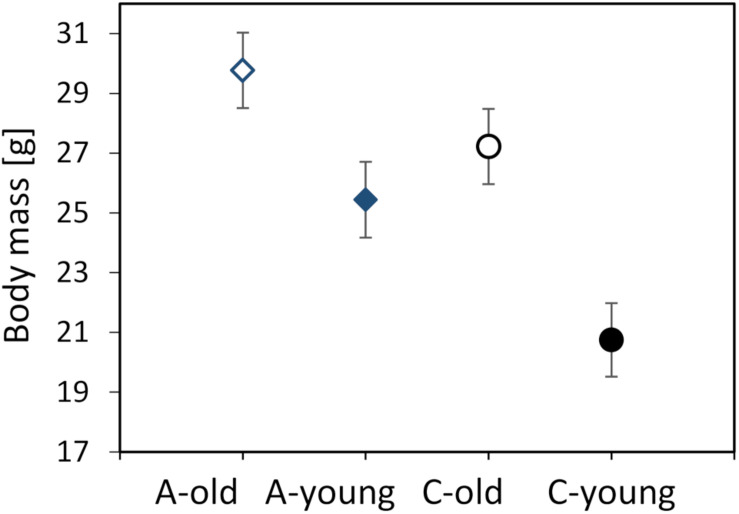
Body mass (in grams) measured before the first respirometric trial of the old (*dashed lines*, *open symbols*) and young (*solid lines*, *closed symbols*) bank voles from the selected (*A*, *blue diamonds*) and control lines (*C*, *black circles*). The *points* represent the adjusted least squares means (LSMs) and *whiskers* represent the standard errors (SEs).

The body mass of the voles, measured before the first respirometric trial, ranged from 15.9 to 37.5 g (mean ± SD = 25.6 ± 5.35 g). The body mass did not differ between the three experimental blocks (*F*_1_,_107_ = 1.40, *p* = 0.25) or between the morning and afternoon trials (*F*_1_,_111_ = 0.88, *p* = 0.35). The mean mass was higher in old voles (LSM ± SE = 28.5 ± 0.89 g) than in young ones (23.1 ± 0.88 g; *F*_1_,_6_._5_ = 51.8, *p* = 0.0003) and tended to be higher in voles from the A lines (27.6 ± 1.14 g) than in those from the C lines (24.0 ± 1.13 g; *F*_1_,_6_._0_ = 5.06, *p* = 0.065; Figure 1). The interaction between the effects of age and selection was not significant (*F*_1_,_6_._3_ = 2.07, *p* = 0.20).

### The Confounding Effects of Activity

In all analyses, the RMR increased with increasing activity index (*p* < 0.05), and the RMR estimated for the modal value of SD = 0.02 was 3–5% higher in comparison to that estimated for SD = 0.05 (value close to SD = 0.06 assumed as border of outliers in the activity criterion) at all *T*_*a*_ values below 32°C. At 32°C, this RMR difference was 7% for the A lines and 8% for the C lines. The effect of the activity index was not significant for *T*_*b*_rmr at ambient temperatures below 32°C (*p* > 0.1), but at 32°C the body temperature increased with the activity index (*p* = 0.04). The activity index did not affect the results of thermal conductance (*p* > 0.15). The EWL increased with increasing activity index at all temperatures except at 20°C (20°C: *p* = 0.57; 32°C: *p* = 0.01; *p* < 0.0001 at other *T*_*a*_ values).

### Resting Metabolic Rate

**FIGURE 2:**
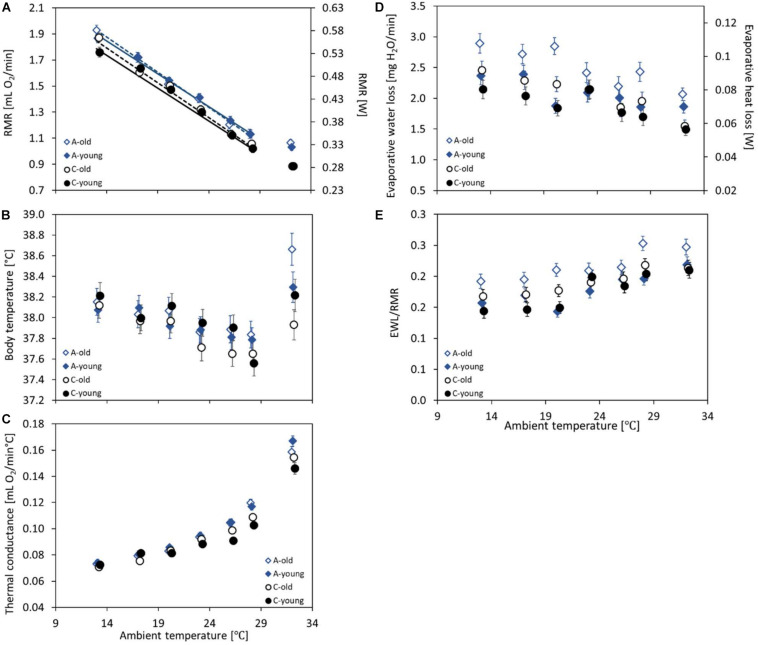
The mass-adjusted resting metabolic rate (RMR, in milliliters O_2_ per minute and *W*) **(A)**, body temperature (*T*_*b*_rmr, in degree Celsius) recorded together with the RMR **(B)**, the thermal conductance (*C*_*T*_, in milliliters O_2_ per minute per degree Celsius) **(C)**, the evaporative water loss (EWL, in milligrams H_2_0 per minute) and its heat loss rate equivalent (*W*) **(D)**, and the EWL/RMR ratio (dimensionless) **(E)** at ambient temperatures (*T*_*a*_) ranging from 13 to 28°C of the old (*dashed lines*, *open symbols*) and young (*solid lines*, *closed symbols*) bank voles from the selected (*A*, *blue diamonds*) and control lines (*C*, *black circles*). The *points* represent the adjusted least squares means (LSMs) for a body mass of 25 g and SD of RMR of 0.03; *whiskers* represent the standard errors (SEs). The regression lines (*A*) are from the repeated-measures ANCOVA model not including the results from 20 or 32°C (see section “Materials and Methods”), with slopes common for the selection groups but different for the age groups.

The RMR measured at 20°C ranged from 1.04 to 2.10 ml O_2_ min^–1^ (mean ± SD = 1.5 ± 0.25 ml O_2_ min^–1^) and increased with body mass (linear slope ± SE = 0.037 ± 0.003 ml O_2_ min^–1^ g^–1^; *F*_1_,_114_ = 149, *p* < 0.0001; Supplementary Figure 2). The RMR tended to be, on average, 3% higher in the morning than in the afternoon (*F*_1_,_114_ = 3.45, *p* = 0.067) and varied among the three experimental blocks (*F*_1_,_114_ = 4.56, *p* = 0.013). Analysis of covariance showed that the mass-adjusted RMR did not differ between age groups (*F*_1_,_114_ = 0.01, *p* = 0.94) or between selection directions (*F*_1_,_114_ = 2.24, *p* = 0.14). The interaction between these factors was also not significant (*F*_1_,_114_ = 1.55, *p* = 0.22; Supplementary Table 3 and Figure 2A).

The repeated-measures analysis of RMR recorded at five ambient temperatures (*T*_*a*_ = 13, 17, 23, 26, and 28°C) revealed that the measurement order affected the results (*F*_4_,_132_ = 3.86, *p* = 0.005), although no systematic trend was observed (the third trial gave the highest results), and that the RMR decreased with increasing *T*_*a*_ from 1.86 ± 0.02 ml O_2_ min^–1^ at 13°C to 1.09 ± 0.02 ml O_2_ min^–1^ at 28°C (*F*_4_,_293_ = 447, *p* < 0.0001; Supplementary Tables 4, 5 and Figure 2A). The resting metabolic rate increased with body mass (linear slope ± SE = 0.029 ± 0.003 ml O_2_ min^–1^ g^–1^; *F*_1_,_766_ = 119, *p* < 0.0001; Supplementary Figure 2) and was higher in the morning than in the afternoon (*F*_1_,_110_ = 5.26, *p* = 0.024). The mass-corrected RMR was, on average, 6% higher in the A lines than in the C lines (A: 1.48 ± 0.02 ml O_2_ min^–1^, C: 1.38 ± 0.02 ml O_2_ min^–1^; *F*_1_,_4_ = 12.7, *p* = 0.023) and did not differ between the two age groups (*F*_1_,_132_ = 0.47, *p* = 0.50; Supplementary Tables 4,5). The interaction between selection direction and age was not significant (*F*_1_,_115_ = 0.41, *p* = 0.53; Supplementary Tables 4, 5 and Figure 2A). Interactions between *T*_*a*_ and age or selection were not significant either (*T*_*a*_ × age: *F*_4_,_304_ = 2.06, *p* = 0.087; *T*_*a*_ × selection: *F*_4_,_306_ = 0.04, *p* = 0.99). The analysis with *T*_*a*_ treated as a quantitative predictor gave practically the same results concerning the main factors. The resting metabolic rate decreased with increasing *T*_*a*_ (-0.052 ± 0.0012 ml O_2_ min^–1^°C^–1^; *F*_1_,_297_ = 1830, *p* < 0.0001). The slope did not differ between selection directions or age groups either (*T*_*a*_ × age: *F*_1_,_342_ = 2.66, *p* = 0.10; *T*_*a*_ × selection: *F*_1_,_339_ = 0.0, *p* = 0.96). The RMR was higher by 0.09 ± 0.02 ml O_2_ min^–1^ in the A than in the C lines (*F*_1_,_342_ = 12.1, *p* = 0.025), but it did not differ between the two age groups (*F*_1_,_342_ = 0.41, *p* = 0.53), and the interaction between selection direction and age was not significant either (*F*_1_,_116_ = 0.48, *p* = 0.49; Figure 2A).

At the highest ambient temperature (32°C), the RMR ranged from 0.53 to 1.67 ml O_2_ min^–1^ and was, on average, lower than that at 28°C (mean ± SD = 0.96 ± 0.21 ml O_2_ min^–1^; Figure 2A). The RMR increased with body mass (linear slope ± SE = 0.021 ± 0.003 ml O_2_ min^–1^ g^–1^; *F*_1_,_44_._4_ = 54.5, *p* < 0.0001; Supplementary Figure 2) and did not differ between the morning and afternoon trials (*F*_1_,_95_ = 1.05, *p* = 0.31). The mass-adjusted RMR was 16% higher in the A than in the C lines (*F*_1_,_5_._5_ = 40.5, *p* = 0.001), but the age effect (*F*_1_,_94_._8_ = 0.47, *p* = 0.50) and the interaction between the factors were not significant (*F*_1_,_95_._5_ = 0.50, *p* = 0.48; Supplementary Table 3 and Figure 2A).

### Body Temperature

Body temperature at the time when the RMR was recorded (*T*_*b*_rmr), measured at the ambient temperature (*T*_*a*_) of 20°C, ranged from 36.3 to 39.4°C (mean ± SD = 38.0 ± 0.6°C). The body temperature decreased with body mass (slope ± SE = -0.025 ± 0.013°C g^–1^; *F*_1_,_85_._8_ = 3.96, *p* = 0.050; Supplementary Figure 3), but did not differ between the morning and afternoon trials (*F*_1_,_125_ = 1.24, *p* = 0.27). The ANCOVA-adjusted least squares means did not differ between age groups (*F*_1_,_1205_ = 0.0, *p* = 0.98) or selection directions (*F*_1_,_6_._6_ = 0.14, *p* = 0.72; Supplementary Table 3 and Figure 2B), and the interaction between the factors was not significant either (*F*_1_,_110_ = 2.02, *p* = 0.16).

The repeated-measures analysis applied to the combined data of *T*_*b*_rmr recorded at five ambient temperatures (*T*_*a*_ = 13, 17, 23, 26, and 28°C) showed a significant effect of the measurement order (*F*_4_,_448_ = 2.47, *p* = 0.04) and no significant effect of the timing (*F*_1_,_115_ = 1.66, *p* = 0.20). The body temperature decreased with increasing ambient temperatures, from 38.1 ± 0.06°C at 13°C to 37.7 ± 0.06°C at 28°C (*F*_4_,_28_._5_ = 11.6, *p* < 0.0001; Supplementary Tables 4, 5 and Figure 2B) and decreased with body mass (slope ± SE = −0.029 ± 0.010°C g^–1^; *F*_1_,_134_ = 7.80, *p* = 0.006; Supplementary Figure 3). The analysis revealed no effect of age (*F*_1_,_15_._9_ = 0.18, *p* = 0.68), selection (*F*_1_,_14_._6_ = 0.57, *p* = 0.46), or their interaction (*F*_1_,_10_._6_ = 0.56, *p* = 0.47). Interactions between *T*_*a*_ and age or selection were not significant either (*T*_*a*_ × age: *F*_4_,_437_ = 0.99, *p* = 0.42; *T*_*a*_ × selection: *F*_4_,_26_._2_ = 0.99, *p* = 0.43).

At the highest ambient temperature (32°C), the *T*_*b*_rmr was markedly higher than those at temperatures of 28 and 26°C (Figure 2B). Unlike in the lower *T*_*a*_ values, at 32°C, the *T*_*b*_rmr did not decrease with body mass (slope ± SE = 0.030 ± 0.018°C g^–1^; *F*_1_,_97_ = 3.06, *p* = 0. 083; Supplementary Figure 3). Again, the *T*_*b*_rmr did not differ between the morning and afternoon trials (*F*_1_,_97_ = 0.16, *p* = 0.69) or between age groups (*F*_1_,_97_ = 0.07, *p* = 0.80). However, the *T*_*b*_rmr was higher in the A than in the C lines (A: 38.5 ± 0.11°C, C: 38.1 ± 0.11°C; *F*_1_,_97_ = 8.12, *p* = 0.005), and the interaction between age and selection direction was also significant (*F*_1_,_97_ = 5.36, *p* = 0.02; Supplementary Table 3). The difference between the selection directions was more profound among the old (A: 38.7 ± 0.15°C, C: 37.9 ± 0.16°C; *F*_1_,_97_ = 14.3, *p* = 0.003) than the young voles (A: 38.3 ± 0.15°C, C: 38.2 ± 0.16°C; *F*_1_,_97_ = 0.15, *p* = 0.70; Figure 2B).

### Thermal Conductance

The thermal conductance (*C*_*T*_) at 20°C ranged from 0.06 to 0.12 ml O_2_ min^–1^°C^–1^ (mean ± SD = 0.08 ± 0.01 ml O_2_ min^–1^°C^–1^) and increased with body mass (linear slope ± SE = 0.0022 ± 0.0002 ml O_2_ min^–1^°C^–1^ g^–1^; *F*_1_,_112_ = 182, *p* < 0.0001; Supplementary Figure 4). As in the case of the *T*_*b*_rmr, the timing effect was not significant (*F*_1_,_121_ = 1.89, *p* = 0.17), but the *C*_*T*_ differed among the three experimental blocks (*F*_2_,_112_ = 6.29, *p* = 0.003). Analysis of covariance revealed that the mass-adjusted *C*_*T*_ did not differ between age groups (*F*_1_,_112_ = 0.206, *p* = 0.80) or between selection directions (*F*_1_,_112_ = 2.32, *p* = 0.13; Supplementary Table 3 and Figure 2C). In the young group, *C*_*T*_ tended to be higher in the A than in the C lines (A: 0.086 ± 0.001 ml O_2_ min^–1^°C^–1^, C: 0.081 ± 0.001 ml O_2_ min^–1^°C^–1^; *F*_1_,_112_ = 5.20, *p* = 0.02), while in the old group the effect of selection was not detectable (LSM averaged across the A and C lines: 0.083 ± 0.001 ml O_2_ min^–1^°C^–1^; *F*_1_,_112_ = 0.02, *p* = 0.89), but the interaction between these factors was not significant (*F*_1_,_112_ = 3.07, *p* = 0.083; Figure 2C).

The repeated-measures analysis of *C*_*T*_ measured at five ambient temperatures (*T*_*a*_ = 13, 17, 23, 26, and 28°C) showed a significant effect of the measurement order (*F*_4_,_438_ = 2.89, *p* = 0.022), but no systematic trend was observed. Thermal conductance increased with increasing *T*_*a*_, from 0.073 ± 0.002 ml O_2_ min^–1^°C^–1^ at 13°C to 0.112 ± 0.002 ml O_2_ min^–1^°C^–1^ at 28°C (*F*_4_,_24_._5_ = 207, *p* < 0.0001; Supplementary Tables 4, 5 and Figure 2C) and increased with body mass (slope ± SE = 0.002 ± 0.00020 ml O_2_ min^–1^°C^–1^ g^–1^; *F*_1_,_99_._7_ = 108, *p* < 0.0001; Supplementary Figure 4). The effects of selection direction and age were complicated by their interactions with *T*_*a*_ (*T*_*a*_ × age: *F*_4_,_435_ = 3.23, *p* = 0.013; *T*_*a*_ × selection: *F*_4_,_22_._3_ = 6.01, *p* = 0.002; Supplementary Tables 4, 5 and Figure 2C). At low ambient temperatures, *C*_*T*_ did not differ between age or selection groups, but with increasing *T*_*a*_ the groups diverged. At high temperatures, *C*_*T*_ was clearly higher in the A than in the C lines (at 28°C, averaged across age groups, A: 0.118 ± 0.002 ml O_2_ min^–1^°C^–1^, C: 0.106 ± 0.002 ml O_2_ min^–1^°C^–1^; *F*_1_,_13_ = 21.4, *p* = 0.0005) and was also higher in the old than in the young voles (at 28°C, old: 0.114 ± 0.002 min^–1^°C^–1^, young: 0.110 ± 0.002 min^–1^°C^–1^; *F*_1_,_376_ = 3.70, *p* = 0.055; Figure 2C).

At 32°C, the thermal conductance was much higher than that at 28°C (Figure 2C). As in lower temperatures, it increased with body mass (linear slope ± SE = 0.0029 ± 0.0005 ml O_2_ min^–1^°C^–1^ g^–1^; *F*_1_,_95_ = 34.2, *p* < 0.0001; Supplementary Figure 4) and did not differ between the morning and afternoon trials (*F*_1_,_95_ = 0.18, *p* = 0.67), but differed among the three experimental blocks (*F*_2_,_95_ = 5.99, *p* = 0.004). The mass-adjusted *C*_*T*_ did not differ significantly between age groups (*F*_1_,_95_ = 0.0, *p* = 0.98), but it was higher in the A than in the C lines (averaged across age groups, A: 0.163 ± 0.003 ml O_2_ min^–1^°C^–1^, C: 0.150 ± 0.003 ml O_2_ min^–1^°C^–1^; *F*_1_,_95_ = 10.2, *p* = 0.002). However, the interaction between age and selection was also significant because in the C lines old individuals tended to have a higher *C*_*T*_, whereas in the A lines the pattern was the opposite (*F*_1_,_95_ = 4.58, *p* = 0.035; Supplementary Table 3 and Figure 2C). Consequently, *C*_*T*_ was much higher in the A than in the C lines in the young group (A: 0.167 ± 0.004 ml O_2_ min^–1^°C^–1^, C: 0.146 ± 0.004 ml O_2_ min^–1^°C^–1^; *F*_1_,_95_ = 13.1, *p* = 0.005), but not in the old group (A: 0.159 ± 0.004 ml O_2_ min^–1^°C^–1^, C: 0.154 ± 0.004 ml O_2_ min^–1^°C^–1^; *F*_1_,_95_ = 0.65, *p* = 0.42; Figure 2C).

### Evaporative Water Loss

The EWL at 20°C ranged from 0.95 to 4.25 mg H_2_O min^–1^ (mean ± SD = 2.2 ± 0.72 mg H_2_O min^–1^) and did not change with body mass (linear slope ± SE = 0.007 ± 0.013 mg H_2_O min^–1^ g^–1^; *F*_1_,_116_ = 0.30, *p* = 0.58; Supplementary Figure 5). As in the case of the *T*_*b*_rmr and *C*_*T*_, the timing effect was not significant (*F*_1_,_113_ = 0.42, *p* = 0.52). The mass-adjusted EWL was 26% higher in the old (2.50 ± 0.10 mg H_2_O min^–1^) than in the young voles (1.86 ± 0.09 mg H_2_O min^–1^; *F*_1_,_115_ = 25.4, *p* < 0.0001; Supplementary Table 3 and Figure 2D), and voles from the A lines had a 12% higher EWL than those from the C lines (*F*_1_,_116_ = 6.21, *p* = 0.014). The interaction between age and selection was also significant, and the EWL was much higher in the A than in the C lines in the old group (A: 2.77 ± 0.13 mg H_2_O min^–1^, C: 2.23 ± 0.12 mg H_2_O min^–1^; *F*_1_,_116_ = 11.8, *p* = 0.0008), but not in the young group (A: 1.88 ± 0.12 mg H_2_O min^–1^, C: 1.84 ± 0.12 mg H_2_O min^–1^; *F*_1_,_113_ = 0.06, *p* = 0.81). In addition, the difference in the EWL between age groups was more remarkable in the A lines (*F*_1_,_114_ = 30.0, *p* < 0.0001) than in the C lines (*F*_1_,_115_ = 5.39, *p* = 0.02; Figure 2D).

The repeated-measures analysis of the EWL calculated for five ambient temperatures (*T*_*a*_ = 13, 17, 23, 26, and 28°C) showed no effect of the measurement order (*F*_4_,_423_ = 1.59, *p* = 0.18), but the EWL decreased with increasing *T*_*a*_, from 2.47 ± 0.10 mg H_2_O min^–1^ at 13°C to 1.99 ± 0.10 mg H_2_O min^–1^ at 28°C (*F*_4_,_24_._9_ = 15.2, *p* < 0.0001; Supplementary Tables 4, 5 and Figure 2D). The EWL increased with body mass (slope ± SE = 0.022 ± 0.011 ml O_2_ mg H_2_O min^–1^ g^–1^; *F*_1_,_121_ = 3.92, *p* = 0.050; Supplementary Figure 5). The mass-adjusted EWL was much higher in the old (2.34 ± 0.10 mg H_2_O min^–1^) than in the young groups (2.05 ± 0.09 mg H_2_O min^–1^; *F*_1_,_174_ = 7.78, *p* = 0.006) and was higher in the A lines (2.34 ± 0.10 mg H_2_O min^–1^) than in the C lines (2.05 ± 0.10 mg H_2_O min^–1^; *F*_1_,_6_._4_ = 6.09, *p* = 0.05). However, neither the interaction between age and selection (*F*_1_,_128_ = 1.65, *p* = 0.20) nor the interactions of these factors with *T*_*a*_ were significant (*T*_*a*_ × age: *F*_4_,_434_ = 1.59, *p* = 0.16; *T*_*a*_ × selection: *F*_4_,_22_._5_ = 0.91, *p* = 0.47; Supplementary Tables 4, 5 and Figure 2D).

At 32°C, the evaporative water loss was surprisingly lower than that at 28°C (Figure 2D). It increased with body mass (linear slope ± SE = 0.034 ± 0.0093 mg H_2_O min^–1^ g^–1^; *F*_1_,_75_._9_ = 12.9, *p* = 0.0006; Supplementary Figure 5), but did not differ between the morning and afternoon trials (*F*_1_,_93_._3_ = 0.68, *p* = 0.45). Unlike in lower temperatures, at 32°C, the mass-adjusted EWL did not differ significantly between age groups (*F*_1_,_8_._6_ = 1.61, *p* = 0.24), but it was higher in the A lines (1.96 ± 0.07 mg H_2_O min^–1^) than in the C lines (1.52 ± 0.07 mg H_2_O min^–1^; *F*_1_,_7_._8_ = 20.9, *p* = 0.002). The interaction between age and selection was not significant (*F*_1_,_7_._6_ = 0.64, *p* = 0.45; Supplementary Table 3 and Figure 2D).

The ratio of the evaporative heat loss and the resting metabolic rate (EWL/RMR) at 20°C ranged from 0.09 to 0.35 (mean ± SD = 0.18 ± 0.055) and decreased with body mass (linear slope ± SE = −0.003 ± 0.001; *F*_1_,_117_ = 9.14, *p* = 0.003; Supplementary Figure 6). As in the case of EWL, the timing effect was not significant (*F*_1_,_113_ = 0.03, *p* = 0.86). The EWL/RMR was 24% higher in the old (0.20 ± 0.008) than in the young voles (0.15 ± 0.007; *F*_1_,_116_ = 21.2, *p* < 0.0001; Supplementary Table 3 and Figure 2E), but did not differ between the A and C lines (*F*_1_,_117_ = 2.10, *p* = 0.15). The interaction between age and selection was significant, and the EWL/RMR was higher in the A than in the C lines in the old group (A: 0.22 ± 0.011, C: 0.18 ± 0.010; *F*_1_,_117_ = 7.01, *p* = 0.009), but not in the young group (A: 0.15 ± 0.010, C: 0.16 ± 0.010; *F*_1_,_114_ = 0.25, *p* = 0.62). In addition, the difference in the EWL/RMR ratio between age groups was more profound in the A lines (*F*_1_,_115_ = 25.8, *p* > 0.0001) than in the C lines (*F*_1_,_116_ = 4.07, *p* = 0.05; Figure 2E).

The repeated-measures analysis of the EWL/RMR ratio calculated for five ambient temperatures (*T*_*a*_ = 13, 17, 23, 26, and 28°C) showed no effect of the measurement order (*F*_4_,_448_ = 1.92, *p* = 0.11), but EWL/RMR increased with increasing *T*_*a*_, from 0.17 ± 0.006 at 13°C to 0.22 ± 0.006 at 28°C (*F*_4_,_449_ = 22.2, *p* < 0.0001; Supplementary Tables 4, 5 and Figure 2E). The EWL/RMR ratio decreases with body mass (slope ± SE = −0.002 ± 0.0008; *F*_1_,_103_ = 4.33, *p* = 0.04; Supplementary Figure 6). The EWL/RMR was higher in the old (0.20 ± 0.007) than in the young groups (0.18 ± 0.006) and did not differ significantly between the A and C lines (*F*_1_,_6_._6_ = 2.96, *p* = 0.13). However, neither the interaction between age and selection (*F*_1_,_124_ = 2.75, *p* = 0.10) nor the interactions of these factors with *T*_*a*_ were significant (*T*_*a*_ × age: *F*_4_,_448_ = 1.15, *p* = 0.33; *T*_*a*_ × selection: *F*_4_,_448_ = 1.26, *p* = 0.28; Supplementary Tables 4, 5 and Figure 2E).

At 32°C, the EWL/RMR ratio did not change with body mass (linear slope ± SE = −0.0005 ± 0.0010; *F*_1_,_90_._7_ = 0.24, *p* = 0.62; Supplementary Figure 6), but did not differ between the morning and afternoon trials (*F*_1_,_93_._8_ = 0.01, *p* = 0.91). Unlike in lower temperatures, at 32°C, the EWL/RMR did not differ significantly between age groups (*F*_1_,_12_ = 1.60, *p* = 0.23) and between selection directions (*F*_1_,_10_._9_ = 2.94, *p* = 0.11). The interaction between age and selection was not significant (*F*_1_,_10_._4_ = 1.0, *p* = 0.34; Supplementary Table 3 and Figure 2E).

### The Thermogenic Capacity

**FIGURE 3:**
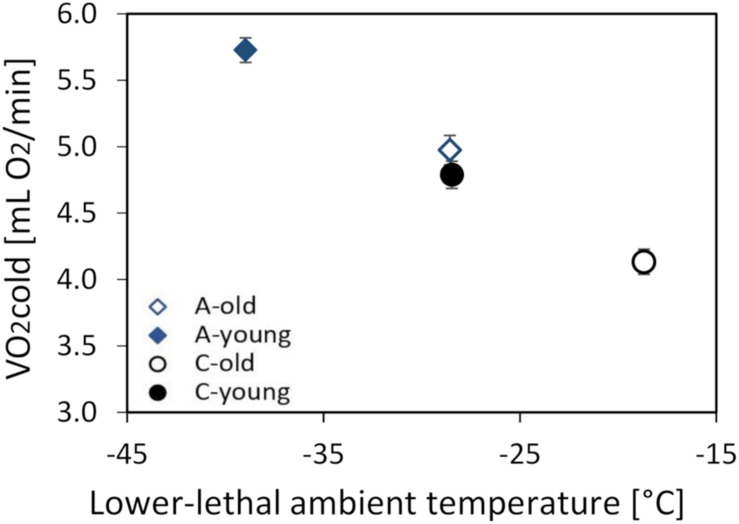
The maximum thermogenesis ($\dot{V}$_*O*__2__*cold*_, in milliliters O_2_ per minute) of the old (*dashed lines*, *open symbols*) and young (*solid lines*, *closed symbols*) bank voles from the selected (*A*, *blue diamonds*) and control lines (*C*, *black circles*), plotted against the lowest temperature they can tolerate. The lower lethal temperature (LLT) was estimated based on the $\dot{V}$_*O*__2__*cold*_ and *C*_*T*_ measured at 13°C. The *points* represent the adjusted least squares means (LSMs) for a body mass of 25 g; *whiskers* represent the standard errors (SEs).

The thermogenic capacity ($\dot{V}$_*O*__2__cold_) ranged from 2.88 to 7.10 ml O_2_ min^–1^ (mean ± SD = 4.86 ± 0.93 ml O_2_ min^–1^), and it increased with body mass (linear slope ± SE = 0.106 ± 0.0113 ml O_2_ min^–1^ g^–1^; *F*_1_,_87_ = 88.3, *p* < 0.0001). The mean values obtained by the two respirometric systems differed by 4% (*F*_1_,_88_._1_ = 6.52, *p* = 0.012), but, as the animals from all groups were randomly assigned to the two systems, the difference had no bearing on the conclusions. The mass-adjusted $\dot{V}$_*O*__2__*cold*_ was 13% lower in the old (4.55 ± 0.07 ml O_2_ min^–1^) than in the young voles (5.26 ± 0.07 ml O_2_ min^–1^; *F*_1_,_9_ = 43.5, *p* < 0.0001; Supplementary Table 3 and Figure 3), and voles from the A lines had a 17% higher $\dot{V}$_*O*__2__*cold*_ than those from the C lines (*F*_1_,_6_._8_ = 74.8, *p* < 0.0001). However, the interaction between these factors was not significant (*F*_1_,_5_._8_ = 0.26, *p* = 0.63; Figure 3).

The lower lethal temperature (LLT) calculated for a 25-g vole was lower in the A than in the C lines and higher in the old than in the young voles, but the difference between age groups was similar in the A and C lines (A lines: 10.4°C, C lines: 9.8°C; Figure 3).

## Discussion

Before discussing the results in detail, we will consider the aspects of the experiment design that had a bearing on the results and limited the scope of inferences. Based on our previous study, we expected that bank voles from the selection experiment should have the thermoneutral zone (TNZ) in the range of temperatures from about 26 to 31°C and that the trials near and above the upper critical temperature (UCT; the upper boundary of TNZ) are associated with an increased risk of mortality (Stawski et al., 2017). We also expected that the repeated trials performed on the same individuals can affect the observed level of metabolism. Therefore, we split the measurements into three stages. Firstly, we measured the resting metabolic rate (RMR) and associated thermoregulatory traits at 20°C, i.e., under the normal housing temperature. The measurements allowed comparisons among the selection and age groups, but they primarily served to habituate the animals to the respirometric trial conditions and, hence, minimize the effect of novelty in the main set of the trials. Secondly, we applied the scheme of a randomized repeated measurers at ambient temperatures (*T*_*a*_) ranging from 13 to 28°C, designed to analyze the relationship between the traits and ambient temperature and to detect differences between the experimental groups in the lower critical temperature (LCT; the lower boundary of TNZ). Finally, to compare the performance under extreme thermal conditions, we measured the thermogenic capacity (the maximum cold-induced metabolic rate) and the RMR (and the associated traits) at 32°C, i.e., at the temperature above the expected UTC. However, it has turned out that the RMR decreased steadily with increasing ambient temperatures and was lower at 32°C than at 28°C, with no evidence of the thermoneutral zone (Figure 2). This inconsistence with the results of our previous study was probably due to the different scheme of the measurements. Previously, the measurements around the thermoneutral zone (25–34°C) were performed in a single trial, with temperatures rising in 3°C increments: 25, 28 (1 h each), 31, and 34°C (0.5 h each) (Stawski et al., 2017). Thus, the duration of the records at each temperature was short. This could contribute to overestimating the RMR measured at these temperatures, and consequently, the estimate of the lower critical temperature could be downwardly biased. Here, the measurements at each temperature were performed as independent trials, and the RMR at 32°C was about 10% lower than that reported by Stawski et al. (2017) for 28–31°C. However, even if the estimates of RMR obtained in the current work can be considered as more reliable, the results cannot be used to estimate the lower critical temperature because the trial at 32°C was not a part of the randomized repeated-measures design. Therefore, we could not achieve one of the intended objectives, i.e., to learn how the selection and age affect the lower boundary of the thermoneutral zone.

The slope of the thermoregulatory curve, within *T*_*a*_ range of 13–28°C, did not differ between the selection and age groups (Figure 2A). Independently of *T*_*a*_, the RMR was higher in the selected A lines than in the control C lines, which indicates that the increased cost of basal metabolism in the A-line voles is added to the thermoregulatory costs. However, the RMR did not differ between age groups. Deep body temperature (*T*_*b*_), measured simultaneously with the RMR, did not differ significantly between the experimental groups, but it increased nearly linearly with decreasing *T*_*a*_ (Figure 2B). The standard model of thermoregulation, expanded to consider separately the deep body (core) and skin surface temperatures, leads to a prediction that if the skin surface temperature is to be maintained constant over a range of ambient temperatures, the deep body temperature must increase proportionally to the difference between the skin surface and ambient temperatures (Lovegrove et al., 1991). Therefore, our observation indicates that the voles attempted to regulate a constant temperature of body surface (which could be verified in further research by thermovision measurements). Interestingly, the opposite pattern is characteristic in humans, where deep body temperature is maintained at about 36.8°C, whereas the skin temperature, influenced more by skin blood flow and environmental conditions, decreases during cold exposure (Lim et al., 2008). The joint information on the RMR and *T*_*b*_, combined in the coefficient of thermal conductance (*C*_*T*_) calculated separately for each individual and *T*_*a*_, revealed that the characteristics of heat exchange were affected both by the selection and aging (Figure 2C). Although at 13°C *C*_*T*_ was nearly identical in all the groups, it increased and diverged among the experimental groups with increasing *T*_*a*_: at 28°C, the thermal conductance was higher in the A than in the C lines and tended to be higher in the old than in the young voles. Thus, although within this range of *T*_*a*_ body temperature did not differ among the experimental groups, the *C*_*T*_ results signal that, at even higher ambient temperatures, the pattern of thermoregulation will be affected by the selection and age.

Measurements of the rate of evaporative water loss (EWL) revealed a more interesting pattern (Figure 2D). The EWL increased with decreasing *T*_*a*_ and was higher in the A than in the C lines, which presumably reflects the differences in the rate of oxygen consumption and, hence, in the rate of the lung’s ventilation and respiratory water loss (see review: Powers et al., 2012; Zieliński and Przybylski, 2012; Minnaar et al., 2014). However, the EWL was also systematically higher in the old than in the young voles, which cannot be explained in this way. One possibility is that the increased water loss of the aged voles is due to an increased lung ventilation rate, compelled by a decreased efficiency of oxygen extraction (Roman et al., 2016; Melamed et al., 2019). The conjecture is strengthened by an even more pronounced difference between the age groups in the EWL/RMR ratio (Figure 2E). Importantly, this tendency was stronger in the A than in the C lines, although the interaction effect was significant only at 20°C. Thus, if this supposition is correct, the selection resulted in accelerating the aging process. Another possibility is that the body surface of old voles is more permeable to water vapor (Haas et al., 2016). In humans, an age-related decline in sweating was reported (Hirata et al., 2015), but sweating is not a primary mechanism for thermoregulation in rodents (Wanner et al., 2015). On the other hand, the insensible water loss through skin and respiration increases with age, both in humans and mice (Dmitrieva and Burg, 2011; Hurlow and Bliss, 2011; Haas et al., 2016). Irrespective of whether the increased EWL is due to a decreased efficiency of the lung’s ventilation or an increased skin permeability, the higher EWL/RMR ratio in the old group indicates an increased proportion of evaporative heat loss. Thus, although within the range of moderate ambient temperatures, thermoregulation appears to pose no challenge to the voles irrespective of their age, the differences in thermal conductance, and, especially, in the rate of evaporative water loss, indicating that aging can adversely affect the performance of voles under more extreme temperatures. Moreover, the differences in the EWL/RMR ratio suggest that voles from the selected lines can be more vulnerable to the adverse effects of aging.

At 32°C, the highest *T*_*a*_ analyzed, *T*_*b*_ was higher than that at 28°C (Figure 2B), presumably as a result of overheating. This effect was much larger in the A-line than in C-line voles. The A-line voles also had a higher EWL than did the C-line ones, and hence also a higher evaporative heat loss, but, apparently, not enough to offset the larger heat gain associated with their higher rate of metabolism (Figures 2A,D). It is worth noting, however, that the EWL at 32°C has not increased compared to that at 28°C, and actually even tended to be lower than that at 28°C. At high ambient temperatures, animals may face a trade-off between the needs of evaporative cooling to prevent overheating on one side and water conservation to prevent an excess dehydration on the other (Czenze et al., 2020). Our results indicated that at 32°C, the voles gave priority to the water conservation at the cost of sacrificing a strict homeothermia. However, it can be expected that at still higher temperatures or during a longer exposure, the protection against imminent danger of overheating would become the priority. Importantly, however, *T*_*b*_ at 32°C was higher than that at 28°C and was also affected by an interaction between the selection and age effects: in the C lines, *T*_*b*_ tended to be lower in the old than in the young voles, but in the A lines the difference was reversed. Similarly, *C*_*T*_ was higher in the old than in the young voles in the C lines, whereas in the A lines *C*_*T*_ was lower in the old than in the young voles (Figure 2C). Thus, the selection for high aerobic exercise performance resulted in a compromised coping with the hot environment by aged voles, even though aging had no such adverse effect in the control lines.

The ability to tolerate cold temperatures declines with age (DeGroot and Kenney, 2007), which is at least partly attributable to a decreased capacity of shivering thermogenesis, resulting from a loss of skeletal muscle mass (Zillikens et al., 2017). Therefore, we hypothesized that the selection for increased aerobic exercise metabolism can attenuate the adverse effects of aging on cold response performance. The results of this work confirmed our earlier findings (Dheyongera et al., 2016; Stawski et al., 2017) that the thermogenic capacity ($\dot{V}$_*O*__2__*cold*_) is higher in voles from the selected lines than in those from the control lines (Figure 3). As we expected, $\dot{V}$_*O*__2__*cold*_ was lower in the old than in the young voles, but the difference was similar in both selection directions. Thus, although both the selection and aging affected thermogenic performance, the selection had not affected the rate of aging. Nevertheless, old voles from the A lines had as high the thermogenic capacity and cold tolerance as the young voles from the C lines (Figure 3). Similarly, the lower lethal temperature (LLT) calculated based on the thermogenic capacity and thermal conductance was lower in the young than in the old group and lower in the A than in the C lines (Figure 3). The differences between age groups were similar in both selection directions, and the LLT predicted for old A-line voles was very close to the LLT predicted for young C-line voles. Thus, the selected voles maintained the thermoregulatory performance expected for a young non-selected adult vole even at the age of nearly 2 years, e.g., exceeding the maximum observed in free-living voles (Petrusewicz, 1983; Rudolf et al., 2017).

To summarize, our results support the hypothesis that the selection for high aerobic exercise metabolism effectively attenuates the adverse effects of aging on cold tolerance. This advantage, however, is traded off by a compromised coping with hot conditions. The heat dissipation limit (HDL) theory provides a conceptual perspective on the evolution of life histories in relation to maximum capacity to dissipate body heat in endotherms (Speakman, 2005; Speakman and Król, 2010). Our findings demonstrate that the heat dissipation limit can differently act on animals at different ages and with a different level of metabolic rate. In the context of the now well-recognized worldwide global warming (Hu et al., 2017), understanding how the rate of metabolism affects age-related changes of thermoregulation is important both from the eco-evolutionary perspective and from the point of view of epidemiological studies and gerontology. An old age is already a widely recognized risk factor for humans during both heat waves and cold spells (Kenney and Munce, 2003). Our study demonstrates that the individual variation in metabolic rate should also be considered in the assessment of the risk for a particular person. Particularly, a high RMR in elderly people should be treated as a marker of increased susceptibility to the adverse effects of overheating, especially because a high RMR also indicates a generally lower health status in the elderly (Schrack et al., 2014). Therefore, the arsenal of standard diagnostic tools oriented for personalized medicine should be extended to also include the measurements of the metabolic rate.

## Data Availability Statement

All datasets presented in this study are included in the article/Supplementary Material.

## Ethics Statement

The animal study was reviewed and approved by II Local Bioethical Committee in Kraków, Poland.

## Author Contributions

ES and MG conceived the experiment. MG performed the experiment and carried out all the measurements. MG, PK, and ES analyzed the data. MG and PK wrote the manuscript with the assistance of ES and UB. All authors commented on the manuscript.

## Conflict of Interest

The authors declare that the research was conducted in the absence of any commercial or financial relationships that could be construed as a potential conflict of interest.
