# Supplementary material for: Age-Related Changes in the Thermoregulatory Properties in Bank Voles From a Selection Experiment
Source: Front Physiol. 2020 Nov 19;11:576304. doi: 10.3389/fphys.2020.576304 (PMC7711078; doi:10.3389/fphys.2020.576304)
Supplement: Supplementary file 1 [file Table_1.pdf]

Supplementary Material

Supplementary Figures

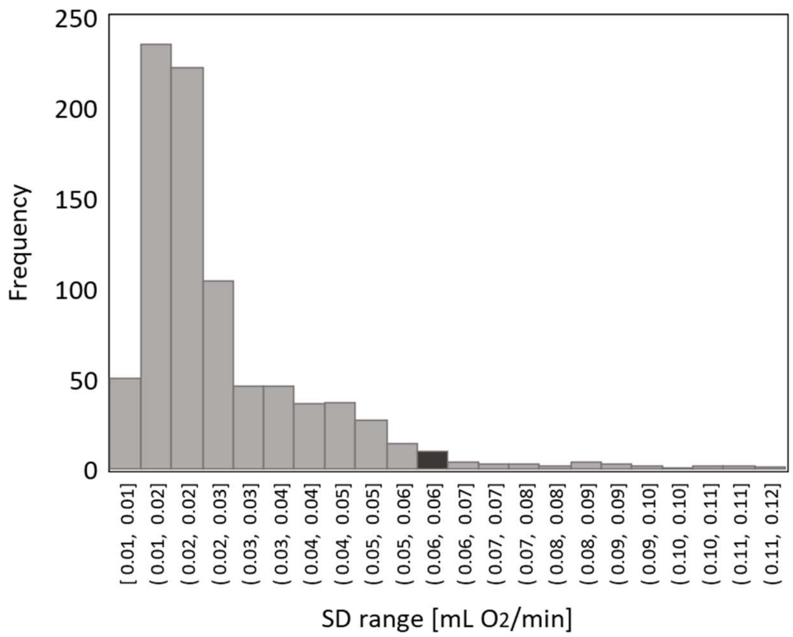

FIGURE S1| Histogram of distribution of the standard deviation (SD) of resting metabolic rate [*RMR;* mlO_2 min^{-1}] used as an activity index. The distribution showed that records with SD over 0.06 mlO_2 min^{-1} (border marked as a dark bin) are outliers from the typical distribution, and these active individuals were excluded from analyses.

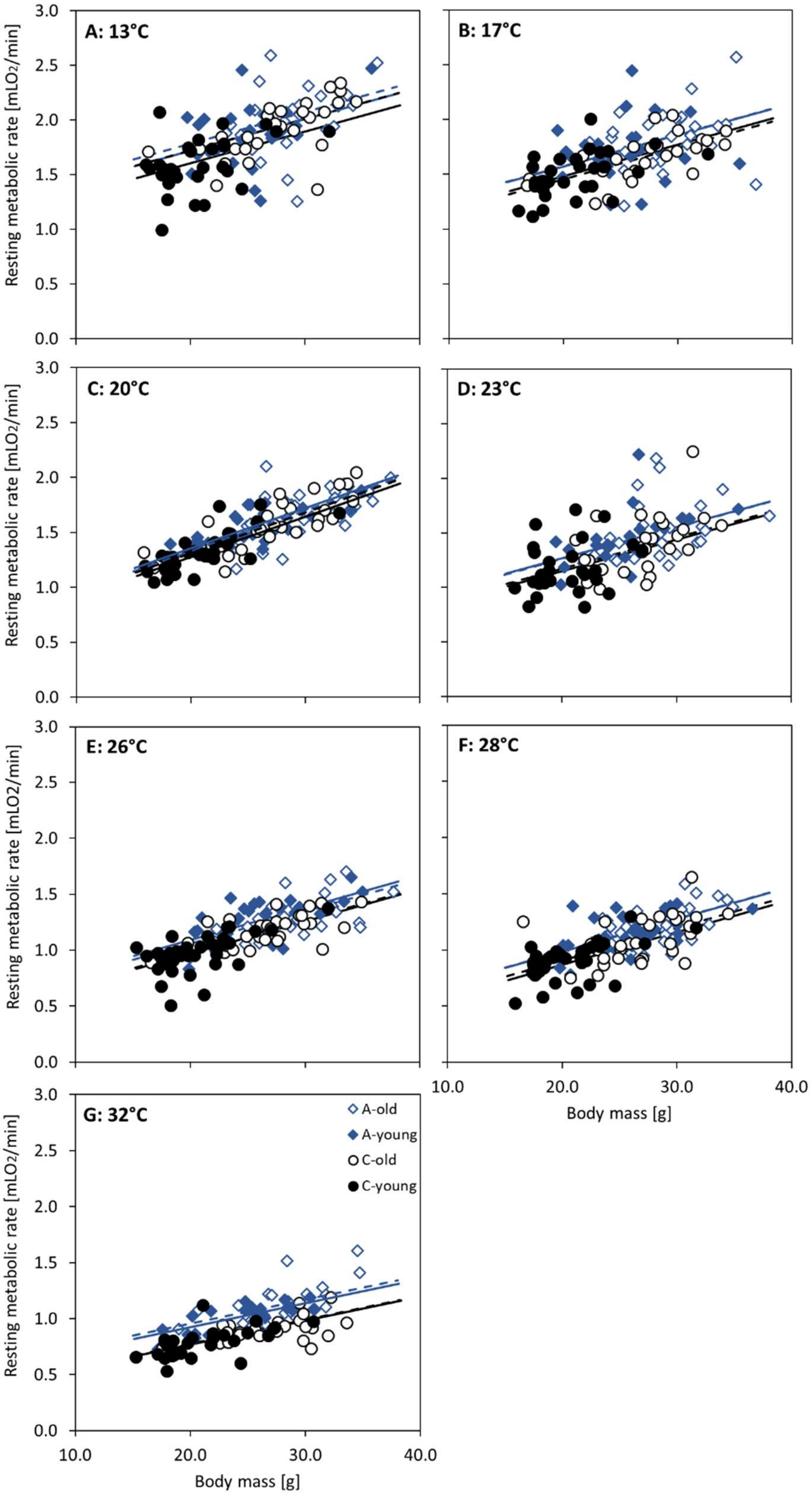

FIGURE S2| The resting metabolic rate [*RMR;* mlO_2 min^{-1}] plotted against body mass [g] at ambient temperatures (T_a): 13, 17, 20, 23, 26, 28, 32 °C (**A-G**) of old (dashed lines, open symbols) and young (solid lines, closed symbols) bank voles from the selected (A – blue diamonds) and control lines (C – black circles). The regression lines are from ANCOVA models.

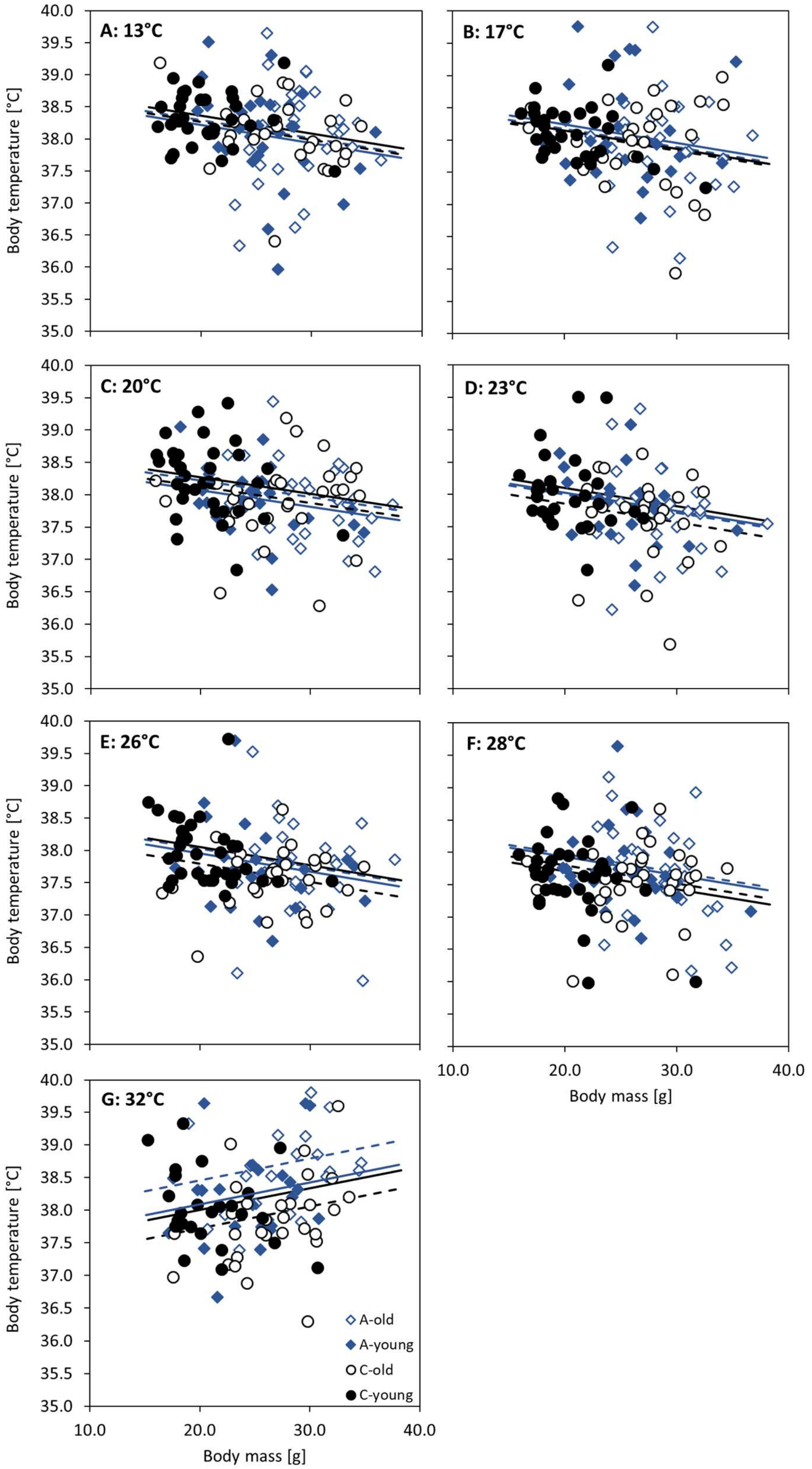

FIGURE S3| The body temperature [$T_b rmr$; °C] recorded together with RMR plotted against body mass [g] at ambient temperatures (T_a): 13, 17, 20, 23, 26, 28, 32 °C (**A-G**) of old (dashed lines, open symbols) and young (solid lines, closed symbols) bank voles from the selected (A – blue diamonds) and control lines (C – black circles). The regression lines are from ANCOVA models.

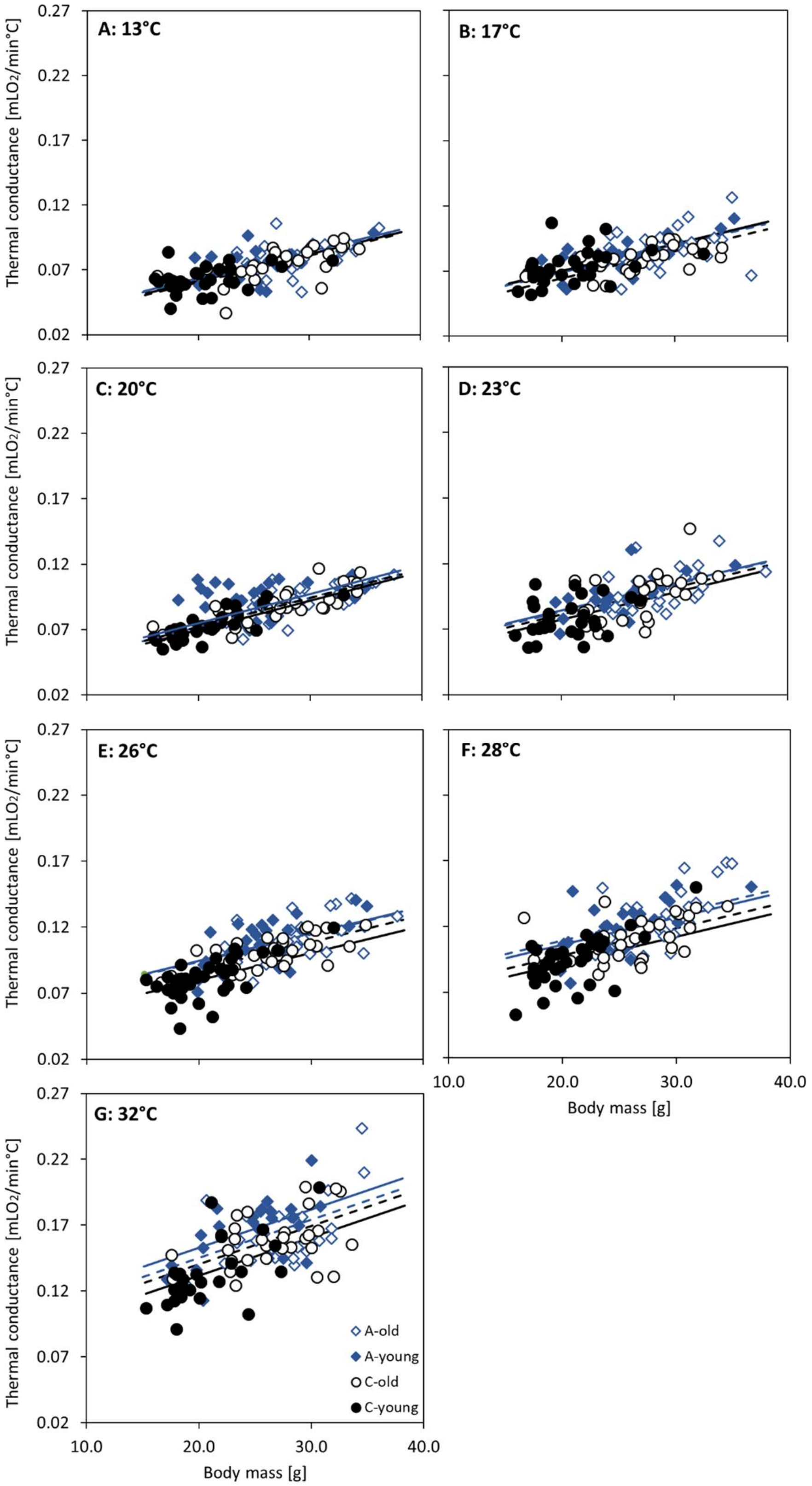

FIGURE S4| The thermal conductance [*CT;* mlO_2 min^{-1} °C^{-1}] plotted against body mass [g] at ambient temperatures (T_a): 13, 17, 20, 23, 26, 28, 32 °C (**A-G**) of old (dashed lines, open symbols) and young (solid lines, closed symbols) bank voles from the selected (A – blue diamonds) and control lines (C – black circles). The regression lines are from repeated measures ANCOVA models.

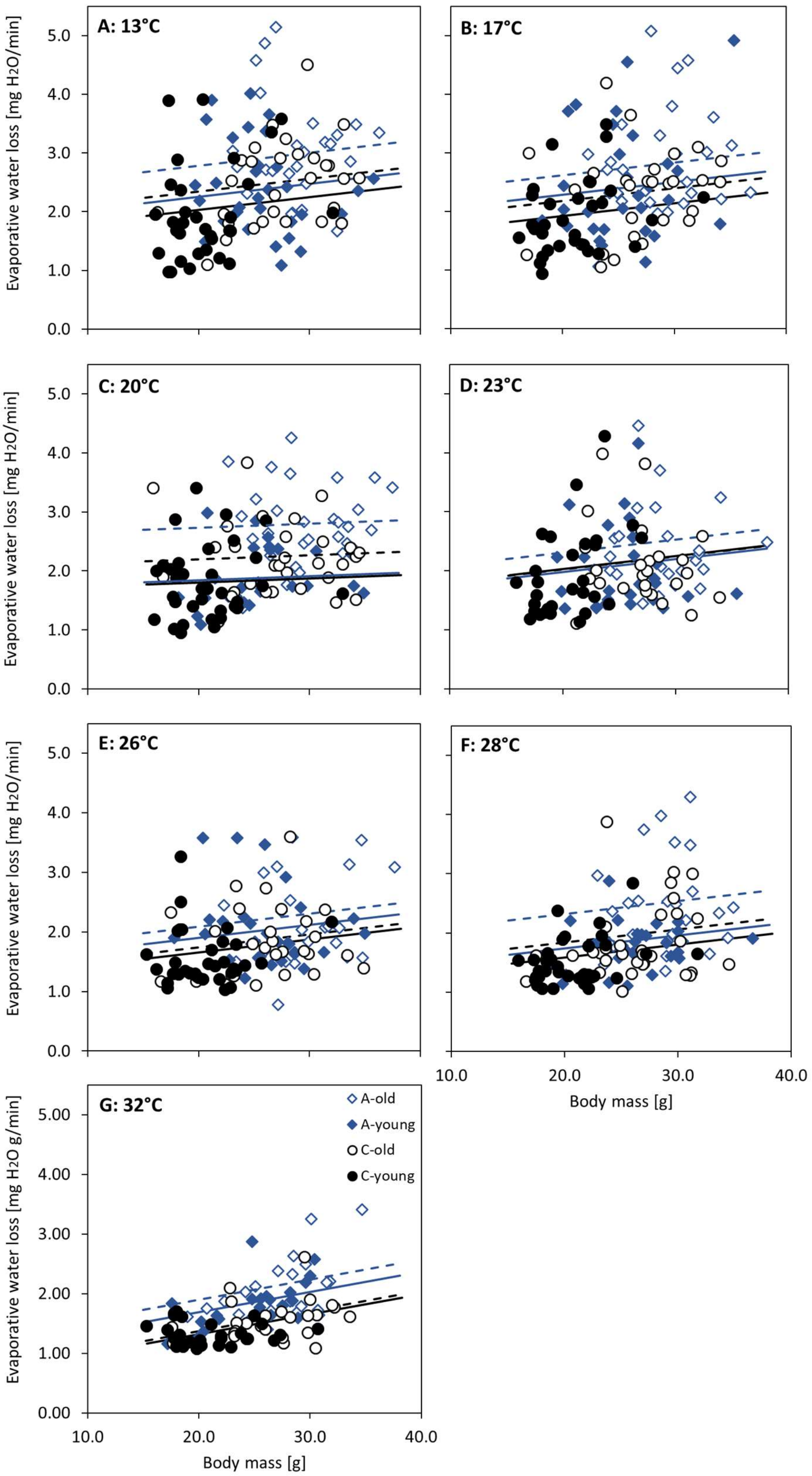

FIGURE S5| The evaporative water loss [*EWL;* mg H20 min^{-1}] plotted against body mass [g] at ambient temperatures (T_a): 13, 17, 20, 23, 26, 28, 32 °C (**A-G**) of old (dashed lines, open symbols) and young (solid lines, closed symbols) bank voles from the selected (A – blue diamonds) and control lines (C – black circles). The regression lines are from ANCOVA models.

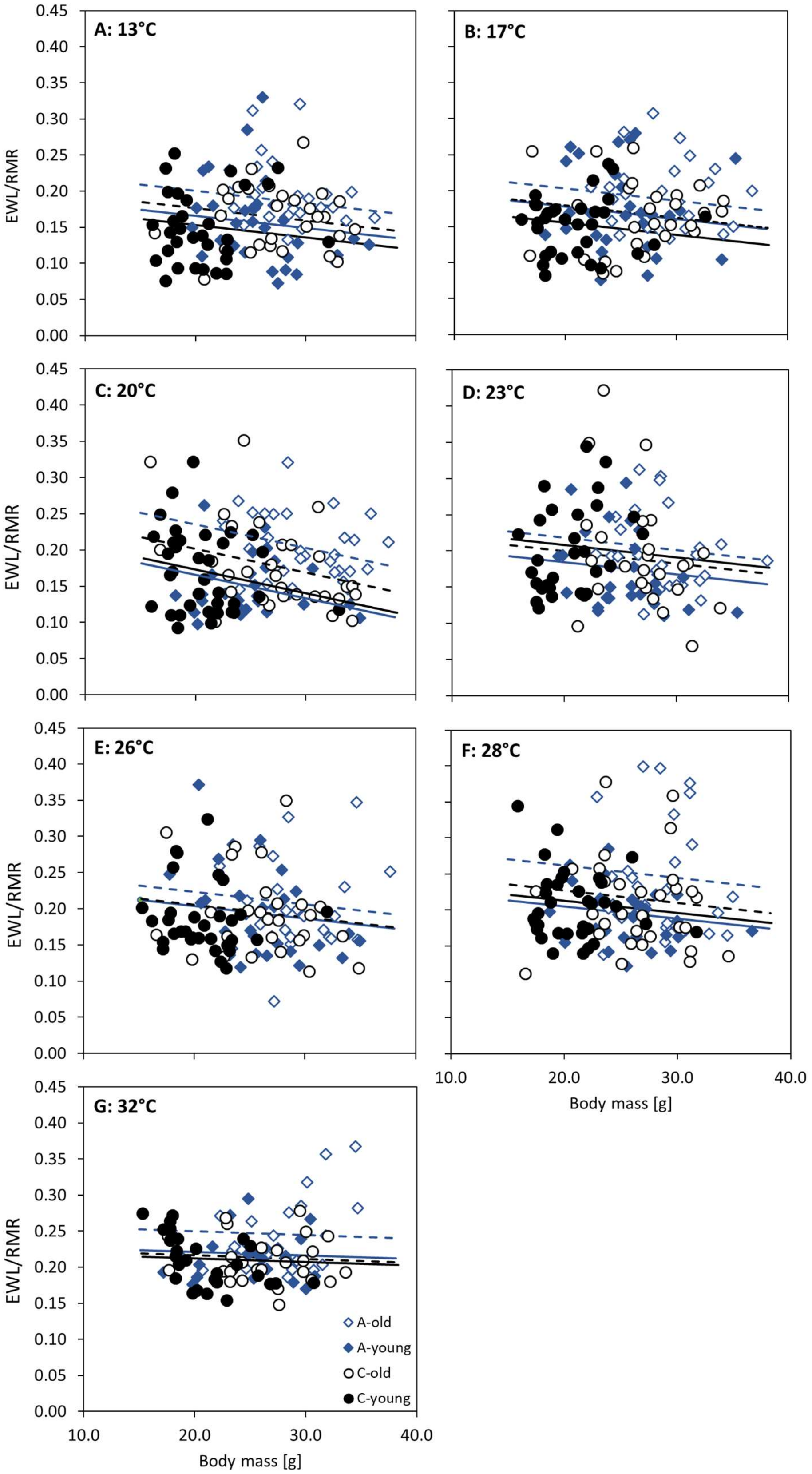

FIGURE S6| The evaporative water loss and resting metabolic rate (*EWL/RMR*) ratio plotted against body mass [g] at ambient temperatures (T_a): 13, 17, 20, 23, 26, 28, 32 °C (**A-G**) of old (dashed lines, open symbols) and young (solid lines, closed symbols) bank voles from the selected (A – blue diamonds) and control lines (C – black circles). The regression lines are from ANCOVA models.

Supplementary Tables

TABLE S1| The order of measurements performed at temperatures: 13, 17, 23, 26 and 28 °C, for each experimental block (A, B, C) and each subgroup of animals measured in the morning or in the afternoon timing.

Experimental block	Animals group + Timing	Order number				
		2.	*3.*	*4.*	*5.*	*6.*
A	1_morning	26 °C	13 °C	17 °C	23 °C	28 °C
	1_afternoon	28 °C	13 °C	23 °C	17 °C	26 °C
	2_morning	17 °C	28 °C	13 °C	26 °C	23 °C
	2_afternoon	23 °C	26 °C	17 °C	28 °C	13 °C
	3_morning	23 °C	13 °C	26 °C	28 °C	17 °C
	3_afternoon	13 °C	17 °C	28 °C	26 °C	23 °C
B	1_morning	26 °C	13 °C	17 °C	23 °C	28 °C
	1_afternoon	28 °C	17 °C	13 °C	23 °C	26 °C
	2_morning	23 °C	28 °C	13 °C	26 °C	17 °C
	2_afternoon	23 °C	26 °C	17 °C	28 °C	13 °C
	3_morning	17 °C	13 °C	26 °C	28 °C	23 °C
	3_afternoon	13 °C	17 °C	28 °C	26 °C	23 °C
C	1_morning	26 °C	13 °C	17 °C	23 °C	28 °C
	1_afternoon	28 °C	17 °C	13 °C	23 °C	26 °C
	2_morning	23 °C	28 °C	13 °C	26 °C	17 °C
	2_afternoon	23 °C	26 °C	17 °C	28 °C	13 °C
	3_morning	17 °C	13 °C	26 °C	28 °C	23 °C
	3_afternoon	13 °C	17 °C	28 °C	26 °C	23 °C

TABLE S2| Summary of number of the observations (with activity index < 0.06) taken for analyses, and number of records removed from final analyses (with studentized residuals higher than 3.5 and lower than -3.5) of the resting metabolic rate [*RMR*, $mlO_2\ min^{-1}$], the body temperature recorded at or near the time *RMR* [$T_b rmr$, °C], the thermal conductance [*CT*, $mlO_2\ min^{-1}\ °C^{-1}$], the evaporative water loss [*EWL*, mg $H_20\ min^{-1}$], *EWL/RMR* ratio for each set of measurements (at 20 °C, 13-28 °C, 32 °C) and for the maximum thermogenesis [VO_2cold, $mlO_2\ min^{-1}$].

Trial	Variable	Number of records	
		Records taken to analyses	*Outliers from analyses*
**20 °C**	***RMR***	125	2
	$T_b rmr$	123	0
	CT	123	2
	EWL	125	2
	EWL/RMR	125	0
**13 - 28 °C**	***RMR***	601	3
	$T_b rmr$	595	2
	CT	595	6
	EWL	594	3
	EWL/RMR	594	1
**32 °C**	***RMR***	108	3
	$T_b rmr$	105	1
	CT	105	1
	EWL	108	0
	EWL/RMR	108	3
	VO_2cold	127	3

TABLE S3| Adjusted least squares means (LSM) ± standard error (SE) and results of ANCOVA of body mass before trial [*MB0,* g], resting metabolic rate [*RMR*, mlO_2 min^{-1}], body temperature recorded at or near the time *RMR* [$T_b rmr$, °C], thermal conductance [*CT*, mlO_2 min^{-1} $°C^{-1}$], evaporative water loss [*EWL,* mg H_20 min^{-1}], *EWL/RMR* ratio measured at the ambient temperature of 20 and 32 °C, and for the maximum thermogenesis [VO_2cold, mlO_2 min^{-1}] and calculated lower lethal temperatures [*LLT,* °C] in old and young bank voles from the selected (A) and control lines (C). *P* values marked with bold if < 0.05.

Trial	Variable	LSM±SE				Significance of effects						
		**Selected (A) old**	**Selected (A) young**	**Control (C) old**	**Control (C) young**	**Selection**	**AgeGr**	**Age* Selection**	**Timing**	**Block**	**Body mass**	**SD**
**20 °C**	***MB0***	29.77±1.26	25.44±1.27	27.22±1.26	20.75±1.23	0.07	**0.0003**	0.20	0.35	0.25	-	-
	RMR	1.51±0.038	1.54±0.026	1.50±0.026	1.47±0.026	0.14	0.94	0.22	**0.07**	**0.01**	**< 0.0001**	**0.04**
	$T_b rmr$	38.07±0.13	37.92±0.12	37.97±0.12	38.11±0.12	0.72	0.98	0.16	0.27	-	**0.05**	0.20
	CT	0.08±0.002	0.09±0.001	0.08±0.001	0.08±0.001	0.13	0.80	0.08	0.17	**0.003**	**< 0.0001**	0.15
	EWL	2.77±0.13	1.88±0.12	2.23±0.12	1.84±0.12	**0.01**	**< 0.0001**	**0.02**	0.52	-	0.58	0.57
	EWL/RMR	0.22±0.010	0.15±0.010	0.18±0.010	0.16±0.010	0.15	**< 0.0001**	**0.02**	0.86	-	**0.003**	0.66
**32 °C**	***RMR***	1.06±0.025	1.03±0.026	0.89±0.025	0.88±0.026	**0.001**	0.50	0.48	0.31	-	**< 0.0001**	**0.02**
	$T_b rmr$	38.66±0.15	38.30±0.15	37.93±0.15	38.22±0.16	**0.005**	0.80	**0.02**	0.69	-	0.08	**0.04**
	CT	0.16±0.004	0.17±0.004	0.15±0.004	0.15±0.004	**0.002**	0.98	**0.04**	0.67	**0.004**	**< 0.0001**	0.44
	EWL	2.07±0.10	1.86±0.10	1.55±0.10	1.50±0.10	**0.002**	0.24	0.45	0.41	-	**0.0006**	**0.03**
	EWL/RMR	0.25±0.013	0.22±0.013	0.21±0.012	0.21±0.013	0.11	0.23	0.34	0.91	-	0.62	0.16
		**Selected (A) old**	**Selected (A) young**	**Control (C) old**	**Control (C) young**	**Selection**	**AgeGr**	**Age* Selection**	**Analyser**	**Body mass**		
	VO_2cold	4.97±0.11	5.73±0.09	4.13±0.09	4.79±0.10	**< 0.0001**	**< 0.0001**	0.63	**0.01**	**< 0.0001**		
	LLT	-28.6	-39.0	-18.7	-28.5	-	-	-	-			

TABLE S4| Results of the repeated-measures analyses for of resting metabolic rate [*RMR*, mlO_2 min^{-1}], body temperature recorded at or near the time *RMR* [$T_b rmr$, °C], thermal conductance [*CT*, mlO_2 min^{-1} $°C^{-1}$], the evaporative water loss [*EWL,* mg H_20 min^{-1}], *EWL/RMR* ratio at ambient temperatures range 13-28 °C of old and young bank voles from the selected (A) and control lines (C). For *RMR* two variables were showed, *RMR(1)*:results of analysis with T_a treated as a categorical predictor, and *RMR(2):* results of analysis with T_a treated as a quantitative predictor. *P* values marked with bold if < 0.05.

Variable	Significance of effects									
	T_a	Selection	AgeGr	Age* Selection	T_a* Selection	T_a*Age	T_a_order	Timing	Body mass	SD
RMR(1)	**< 0.0001**	**0.02**	0.50	0.52	0.99	**0.09**	**0.005**	**0.02**	**< 0.0001**	**0.05**
RMR(2)	**< 0.0001**	**0.03**	0.53	0.49	-	-	**0.007**	**0.01**	**< 0.0001**	**0.02**
$T_b rmr$	**< 0.0001**	0.46	0.68	0.47	0.43	0.42	**0.04**	0.20	**0.006**	0.10
CT	**< 0.0001**	**0.03**	0.56	0.46	**0.002**	**0.01**	**0.02**	0.10	**< 0.0001**	0.23
EWL	**< 0.0001**	**0.05**	**0.006**	0.20	0.47	0.43	0.18	**0.009**	**0.05**	**< 0.0001**
EWL/RMR	**< 0.0001**	0.13	**0.001**	0.10	0.28	0.33	0.11	**0.05**	**0.04**	**0.04**

TABLE S5| Adjusted least squares means (LSM) ± standard error (SE) of old and young bank voles from the selected (A) and control lines (C), for of resting metabolic rate [*RMR*, mlO_2 min^{-1}], body temperature recorded at or near the time *RMR* [$T_b rmr$, °C], thermal conductance [*CT*, mlO_2 min^{-1} $°C^{-1}$], the evaporative water loss [*EWL*, mg H_20 min^{-1}], ratio of evaporative heat loss (*EHL*) and *RMR* (*EHL/RMR*) at ambient temperatures range 13-28 °C.

Trial	Variable	LSM±SE			
		**Selected (A) old**	**Selected (A) young**	**Control (C) old**	**Control (C) young**
**13 °C**	***RMR***	1.93±0.039	1.87±0.036	1.87±0.037	1.76±0.037
	$T_b rmr$	38.15±0.13	38.07±0.12	38.12±0.13	38.22±0.13
	CT	0.07±0.003	0.07±0.003	0.07±0.003	0.07±0.003
	EWL	2.89±0.16	2.37±0.14	2.45±0.15	2.14±0.15
	EWL/RMR	0.19±0.012	0.16±0.011	0.17±0.011	0.14±0.011
**17 °C**	***RMR***	1.72±0.038	1.72±0.034	1.60±0.036	1.64±0.036
	$T_b rmr$	38.03±0.13	38.10±0.12	38.00±0.12	38.00±0.13
	CT	0.08±0.002	0.08±0.002	0.08±0.001	0.08±0.003
	EWL	2.72±0.16	2.39±0.14	2.29±0.15	2.04±0.15
	EWL/RMR	0.19±0.012	0.17±0.012	0.17±0.011	0.15±0.011
**23 °C**	***RMR***	1.40±0.033	1.41±0.031	1.32±0.033	1.30±0.032
	$T_b rmr$	37.86±0.13	37.88±0.13	37.71±0.13	37.95±0.13
	CT	0.09±0.003	0.09±0.003	0.09±0.003	0.09±0.003
	EWL	2.42±0.16	2.09±0.15	2.14±0.16	2.15±0.15
	EWL/RMR	0.21±0.012	0.18±0.011	0.19±0.012	0.20±0.11
**26 °C**	***RMR***	1.20±0.034	1.24±0.031	1.13±0.032	1.12±0.032
	$T_b rmr$	37.89±0.13	37.81±0.12	37.65±0.12	37.90±0.12
	CT	0.10±0.003	0.10±0.003	0.10±0.003	0.09±0.003
	EWL	2.19±0.16	2.01±0.15	1.85±0.15	1.77±0.15
	EWL/RMR	0.21±0.012	0.19±0.011	0.20±0.011	0.18±0.011
**28 °C**	***RMR***	1.13±0.034	1.13±0.031	1.06±0.031	1.02±0.032
	$T_b rmr$	37.83±0.13	37.78±0.12	37.65±0.12	37.56±0.12
	CT	0.12±0.003	0.12±0.002	0.11±0.003	0.10±0.003
	EWL	2.43±0.16	1.86±0.14	1.95±0.15	1.70±0.15
	EWL/RMR	0.25±0.012	0.20±0.010	0.22±0.011	0.20±0.011
